# Development of a closed-loop solvent-based recycling process for dyed denim waste

**DOI:** 10.1039/d6ra04250c

**Published:** 2026-07-18

**Authors:** Md. Reazuddin Repon, Floriane Jacquin, Shubhajit Dutta, Tonmoy Saha, Inge Schlapp-Hackl, Tapani Vuorinen, Ali R. Tehrani-Bagha

**Affiliations:** a Department of Bioproducts and Biosystems, School of Chemical Engineering, Aalto University Vuorimiehentie 1 02150 Espoo Finland ali.tehrani@aalto.fi; b Agro Toulouse, Institute National Polytechnique de Toulouse Auzeville-Tolosane France

## Abstract

The sustainable recycling of indigo-dyed denim waste remains a major challenge due to the chemical stability and strong fiber affinity. In this study, a closed-loop process for the recycling of indigo-dyed denim waste is developed using dimethyl sulfoxide (DMSO) as a recyclable solvent for color stripping and dye recovery. The influence of solvent composition revealed that 100% DMSO, achieved superior indigo extraction compared to aqueous DMSO systems, achieving colour removal efficiencies of up to 98%. Key process parameters, including treatment time, temperature, and material-to-liquor ratio, were optimized to maximize stripping efficiency. Mechanical characterisation revealed excellent preservation of the cellulose substrate, with tensile strength retention of approximately 98%. The stripped denim exhibited high lightness values (L* > 81), making it potentially a good white feedstock for further recycling or re-dyeing. The recovered DMSO was successfully reused for at least five consecutive stripping cycles without a noticeable loss of performance, demonstrating excellent solvent recyclability. This work provides a potential approach for the closed-loop recycling of indigo-dyed denim waste, supporting circular economy principles and contributing to the development of more sustainable textile recycling processes.

## Abstract 2

A DMSO-based process recycles indigo-dyed denim waste through color stripping, solvent recovery, and fiber preservation. Recovered solvent was reused over multiple cycles, and the denim was successfully re-dyed with vat, reactive, and direct dyes.

## Graphical abstract

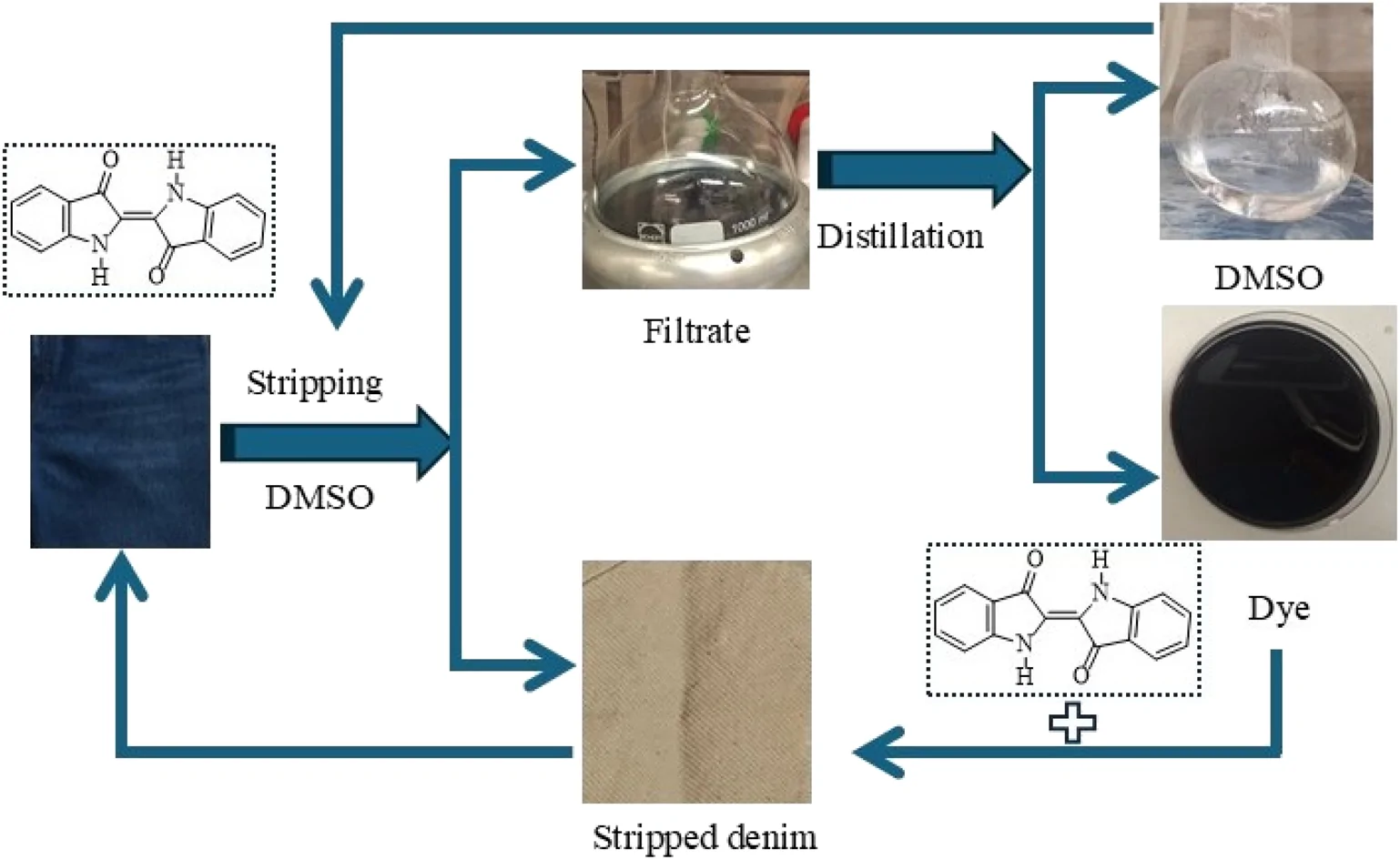

## Introduction

The global textile industry uses large amounts of resources and causes serious environmental problems as it produces high levels of solid waste, wastewater, and chemical pollution at every stage of production.^1,2^ Denim represents one of the largest sub-sectors of this textile and fashion industry, owing to its durability, shade, and comfort, making it an ideal clothing for everyday wear regarding ages, genders, and lifestyles.^3^ The global denim market is projected to grow to 131.3 billion USD by 2032, with the annual denim production exceeding 4 billion pairs.^4,5^ The production of denim is resource and utilities-intensive as it requires 1000 kg of cotton fiber, 802.86 m^3^ of water, and 17.6 MWh of electricity per ton of denim apparel.^6^ The consequences of denim production result in a high amount of carbon dioxide emissions and a huge amount of waste at the end of their life cycle.^7^ The end of life of the denim garments dyed with indigo dyes lacks systematic recycling, thus ending up in landfills or incinerated, which adversely intensifies the environmental pollution.^8^ The incineration of cotton-based denim waste contributes significantly to greenhouse gas emissions, highlighting the need for effective recycling strategies.

Researchers have investigated the recycling route of denim garments, predominantly emphasising downcycling applications such as construction reinforcement, insulation materials, composites, and automotive interiors.^9–11^ However, the upcycling of denim waste to convert into new textiles is still not in practice widely yet. To facilitate this circularity, it is important to address one of the critical challenges in denim recycling, which is the presence of persistent indigo vat dyes.^12,13^ Several chemical approaches have been explored for recycling denim, particularly to address dye removal and fiber recovery; however, each method presents notable limitations.^14–16^ For instance, sequential acid–dithionite–peroxide treatments, involving hydrolysis followed by reduction and oxidation, have been investigated for recovering cellulose from post-consumer denim, but the multistep nature of the process increases chemical consumption, process complexity, and potential cellulose degradation.^15^ Alkali-assisted sodium dithionite reduction has been studied for removing vat dyes and elastane components, yet it requires harsh alkaline conditions and elevated temperatures, raising concerns about fiber damage, chemical safety, and scalability.^16^ Besides, these chemical-based approaches lead to secondary pollution and low environmental sustainability.

Currently, solvent-based treatment has gained attention to remove the dyes from the surface of the fiber,^17^ among which dimethyl sulfoxide (DMSO) is one of the green polar solvents that can be used for color removal.^18^ DMSO is typically classified under GHS as a category 4 flammable liquid, which is combustible, has a boiling and flash point of 189 °C and 870 °C, respectively.^19^ It has strong dissolving ability, low volatility, and high thermal stability.^20^ DMSO also has a better environmental and safety profile than many other solvents such as *N*,*N*-dimethyl formamide, *N*-methyl-2-pyrrolidone, *etc*,.^21^ DMSO can be easily recovered by distillation because of its high boiling point and chemical stability.^22^ Despite these advantages, the potential of DMSO for selective stripping and recovery of indigo dye from post-consumer denim waste has not been thoroughly studied.

Therefore, this study investigates a DMSO-based solvent system for the closed-loop recycling of indigo-dyed denim waste. The proposed approach combines color stripping, solvent recovery and reuse, and preservation of fiber quality within a single process. The effects of DMSO–water composition are examined, and key process parameters, including treatment time, temperature, and material-to-liquor ratio, are optimized to improve color stripping efficiency while reducing solvent and energy demand. A distillation step is employed to recover DMSO and separate the extracted indigo, allowing the recovered solvent to be reused over multiple cycles. In addition, the re-dyeability of the treated denim is evaluated to assess its potential as a feedstock for subsequent textile processing. By integrating dye removal with solvent recycling and fiber preservation, this study contributes to the development of more resource-efficient approaches for recycling indigo-dyed denim waste.

## Experimental

### Materials

The 100% cotton woven fabrics dyed with indigo used in this study were supplied by NZ Fabrics Limited, Bangladesh. The fabric had a 3/1 twill weave structure, with yarn counts of 21/1 Ne in the warp direction and 16/1 Ne in the weft direction. The warp yarns were ring-spun, tightly twisted, and dyed with indigo, whereas the weft yarns were open-end (OE) spun and left undyed (white or grey). No Lycra or spandex fibers were incorporated into the fabric structure. The fabric had a yarn density of 75 ends per cm in the warp and 45 picks per cm in the weft, with an areal density of 310 g m^−2^.

Dimethyl sulfoxide (C_2_H_6_OS, ≥99.9%, CAS No. 67-68-5), sodium dithionite (Na_2_S_2_O_4_, 85%, CAS No. 7775-14-6), and sodium hydroxide (NaOH, 98%, CAS No. 1310-73-2) were purchased from Sigma-Aldrich, Finland. A standard soaping agent (ISO 6330 : 2021, ECE reference detergent 3), free from optical brightening agents and enzymes, was supplied by SDC Enterprises, UK. Commercial indigo powder, Bezathren Blue RS (Vat dye), Bezaktiv Red HP-BL (Reactive dye) and Tubantin Orange GGLN 200 (Direct dye) dyes were obtained from CHT group, Germany. All chemicals were of analytical grade and were used as received, without further purification. DMSO was chosen as the preferred solvent based on Safe and Sustainable by Design (SSbD) framework. In the SSbD scoring system, ranges from 0 (lowest) to 3 (highest), DMSO received a score of 2. This score indicates a high level of safety and environmental sustainability.

### Color stripping process

Prior to the color stripping process, the denim fabric was subjected to an ageing treatment consisting of 10 consecutive washing–drying cycles using a washing machine and a tumble dryer. For each washing cycle, a standard soaping agent was applied under controlled conditions for 1 h at 40 °C, followed by drying for 30 min at 70 °C. This ageing procedure was designed to simulate the cumulative effects of repeated laundering over a fabric's service life, thereby mimicking post-consumer denim waste.

Subsequently, the color stripping process was carried out using an IR sample dyeing machine (Testex TD 130) following the exhaust method. The process parameters, including temperature, treatment time, and material-to-liquor ratio, were varied to optimize the stripping conditions. After stripping, the fabrics were soaped with a standard soaping agent at a concentration of 2 g L^−1^ at 60 °C for 10 min to remove unfixed dye from the fabric surface. A schematic diagram of the solvent-based color stripping process is presented in Fig. S1. Finally, the color-stripped fabrics were oven-dried at 60 °C for 20 min and then conditioned in a standard conditioning room for 24 h prior to characterization.

### Solvent and dye recovery setup

DMSO and indigo dye were recovered by distillation after the color stripping process. First, indigo dyed denim waste was stripped under optimized conditions using fresh DMSO. After stripping, the filtrate contained dissolved DMSO and extracted indigo dyes. This solvent-dye mixture was collected and subjected to distillation to separate the two components.

The distillation setup consists of a heating mantle with round bottom flask that contains filtrate, a condenser (cooling bridge), and a receiving flask (Fig. S2). DMSO has a boiling point of 189 °C at atmospheric pressure. However, DMSO is distilled at approximately 80 °C using 1 mbar pressure. During distillation, DMSO evaporated and condensed in the receiving flasks as purified solvent. The indigo pigments are non-volatile and therefore remained in the original flask. At the end of the process, the pigments formed a concentrated paste. This paste was collected and dried to obtain indigo powder.^23^

### Redyeing of color-stripped denim

Re-dyeing of color-stripped denim fabrics was performed in the TD 130 IR sample dyeing machine using vat (Bezathren Blue RS), reactive (Bezaktiv Red HP-BL), and direct dyes (Tubantin Orange GGLN 200) at a material-to-liquor (M : L) ratio of 1 : 20, with constant agitation.

Vat dye was reduced at 80 °C for 20 min using 10 g L^−1^ sodium dithionite and 3 g L^−1^ sodium hydroxide. Then, stripped denim was added to the bath, and dyeing was carried out at 60 °C for 30 min. After re-dyeing, the fabric was rinsed with overflow water, soaped at 100 °C for 20 min in 2 g L^−1^ ECE reference detergent, rinsed with warm water, and air-dried. In addition, reactive dyeing was carried out at 60 °C for 60 min, containing 30 g L^−1^ Glauber's salt and 20 g L^−1^ sodium carbonate. After re-dyeing, the fabric was rinsed, soaped at 80 °C for 20 min in 2 g L^−1^ ECE reference detergent, rinsed with warm water, and air-dried. Moreover, direct dyeing was conducted at 98 °C for 60 min in the presence of 20 g L^−1^ Glauber's salt and 5 g L^−1^ sodium carbonate. After re-dyeing, the fabric was rinsed, soaped at 40 °C for 20 min in 2 g L^−1^ ECE reference detergent, rinsed with warm water, and air-dried. The re-dyeing process diagram of stripped denim waste using vat (Bezathren Blue RS), reactive (Bezaktiv Red HP-BL), and direct dyes (Tubantin Orange GGLN 200) is shown in Fig. S3.

### Testing and characterization

The colorimetric attributes such as color strength (*K*/*S*), lightness (L*), redness (a*), and blueness (b*) were quantified using an X-Rite Ci7600 spectrophotometer. Measurements were conducted under a D65 illuminant and a 10° standard observer across a spectral range of 360–780 nm. To ensure opacity and measurement consistency, each sample was folded into four layers, and reflectance (*R*) was recorded as the average of five readings at distinct locations. The *K*/*S* were derived *via* the Kubelka–Munk equation (eqn (1)). Color-stripping efficiency was determined through eqn (2), while total color differences were calculated as Delta ECMC (eqn (3)). Furthermore, color fastness to washing and rubbing was evaluated according to ISO 105-C06-A1S:2010 and ISO 105-X12 : 2010, respectively. Changes in shade and staining were assessed using SDC grey scales within a standardized light cabinet. The crystallinity of the denims was investigated *via* X-ray diffraction (XRD) using a Xenocs Xeuss 3.0 diffractometer. The crystallinity index (CI) was calculated using eqn (4).^24^ Surface morphology was visualized using a Zeiss Sigma VP scanning electron microscope (SEM) at an accelerating voltage of 4 kV. Prior to imaging, fabrics were sputter-coated with a gold–palladium (80/20) alloy for 90 seconds to enhance electrical conductivity. Tensile strength was measured in both warp and weft directions using an Instron 4204 universal testing machine, following the ISO 13934-1 strip method. The intrinsic viscosity was determined according to ISO 5351/1 by dissolving denim fabric in cupri-ethylenediamine (CED); these values were used to calculate the degree of polymerization (DP) (eqn (5)). Elemental composition (C, H, N, and S) was analyzed in triplicate using a Thermo Flash Smart CHNS analyzer, calibrated with sulphanilamide standards. Thermogravimetric analysis (TGA) was performed using a Netzsch STA 449 F3 Jupiter system. Denims (6–8 mg) were heated from 40 °C to 600 °C at a constant rate of 10 °C min^−1^ under a nitrogen flow (70 ml min^−1^). For dyeability assessments, dye fixation efficiency was calculated using eqn (6). All denim fabrics were conditioned at 20 ± 2 °C and 66 ± 1% relative humidity prior to testing.1
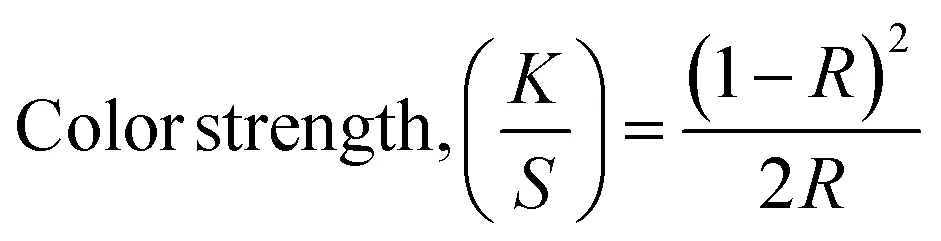
2
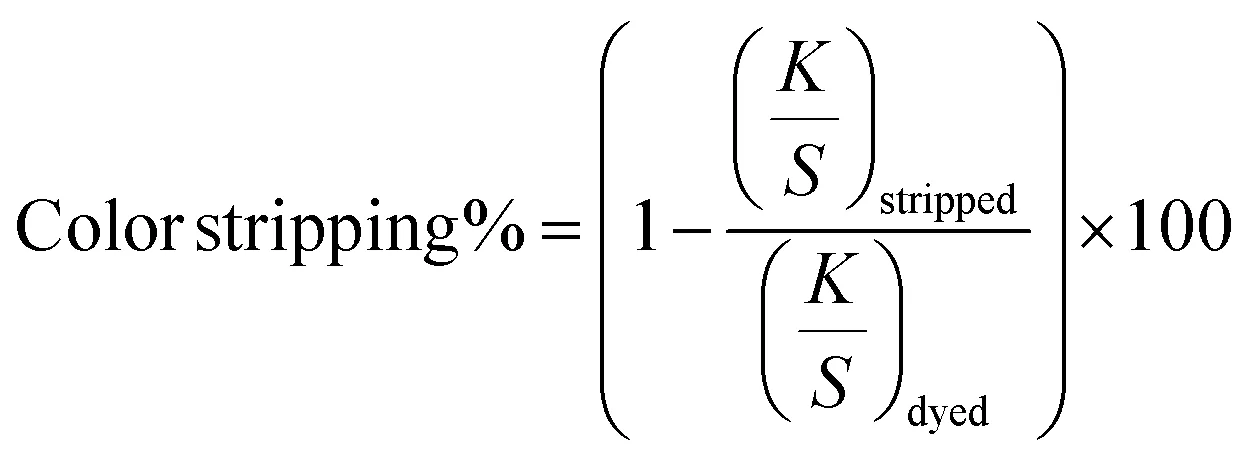
3
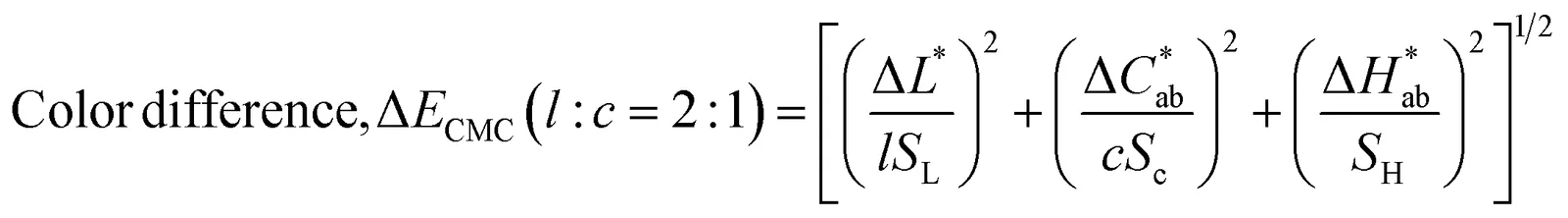
4
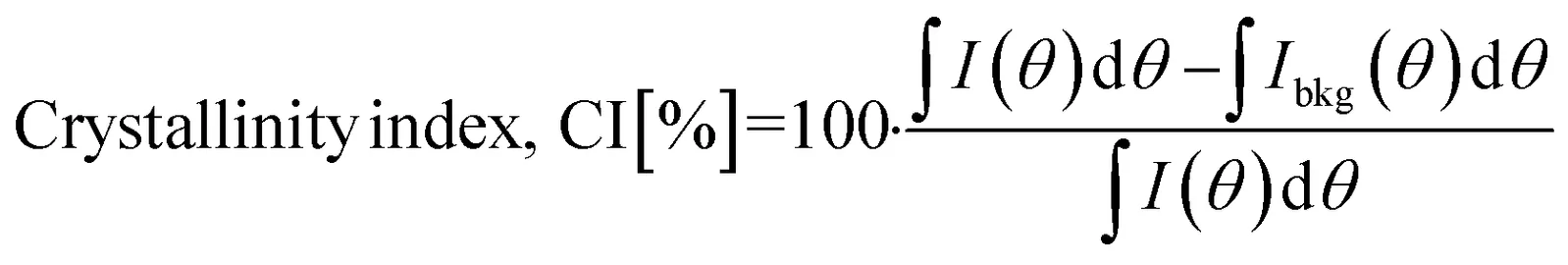

5 Degree of polymerization, DP = 2.381 × Intricsic viscosity

6
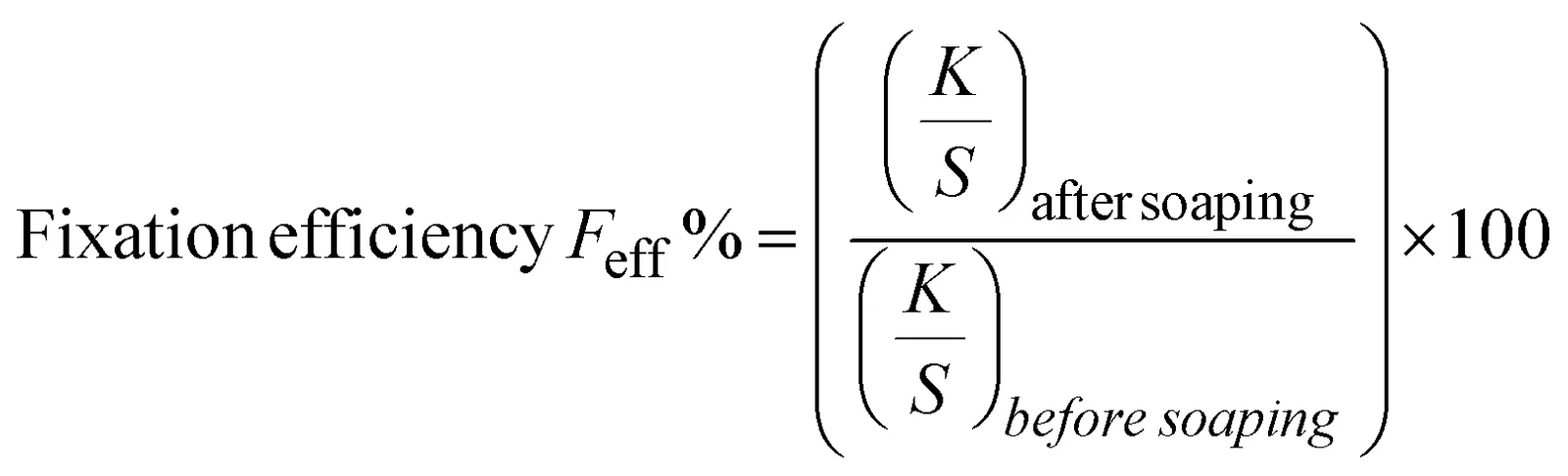


Here, ΔL* = difference in lightness between the sample and the standard; ΔC*_ab_ = difference in chroma between the sample and the standard; ΔH*_ab_ = difference in hue between the sample and the standard; *S*_L_ = weighting functions for lightness; *S*_C_ = weighting functions for chroma; *S*_H_ = weighting functions for hue; *I*(*θ*) = total intensity; *I*_(bkg)_(*θ*) = estimated background intensity.

FT-IR spectra of indigo dye which is recovered after distillation were recorded using a PerkinElmer FT-IR spectrometer equipped with an attenuated total reflectance (ATR) accessory. Measurements were performed over the spectral range of 4000–400 cm^−1^ with a spectral resolution of 4 cm^−1^. Both background and sample spectra were collected using 32 scans.^25^

The ^1^H NMR spectra were obtained using a Bruker Avance III 400 NMR spectrometer and analyzed with MestReNova × 64 software.^26^ A 0.03 mL sample was dissolved in 0.4 mL of DMSO-*d*_6_, and the spectra were referenced to the DMSO-*d*_6_ solvent peak.

## Results and discussion

### Solvent tuning and strategic process optimization

**Fig. 1:**
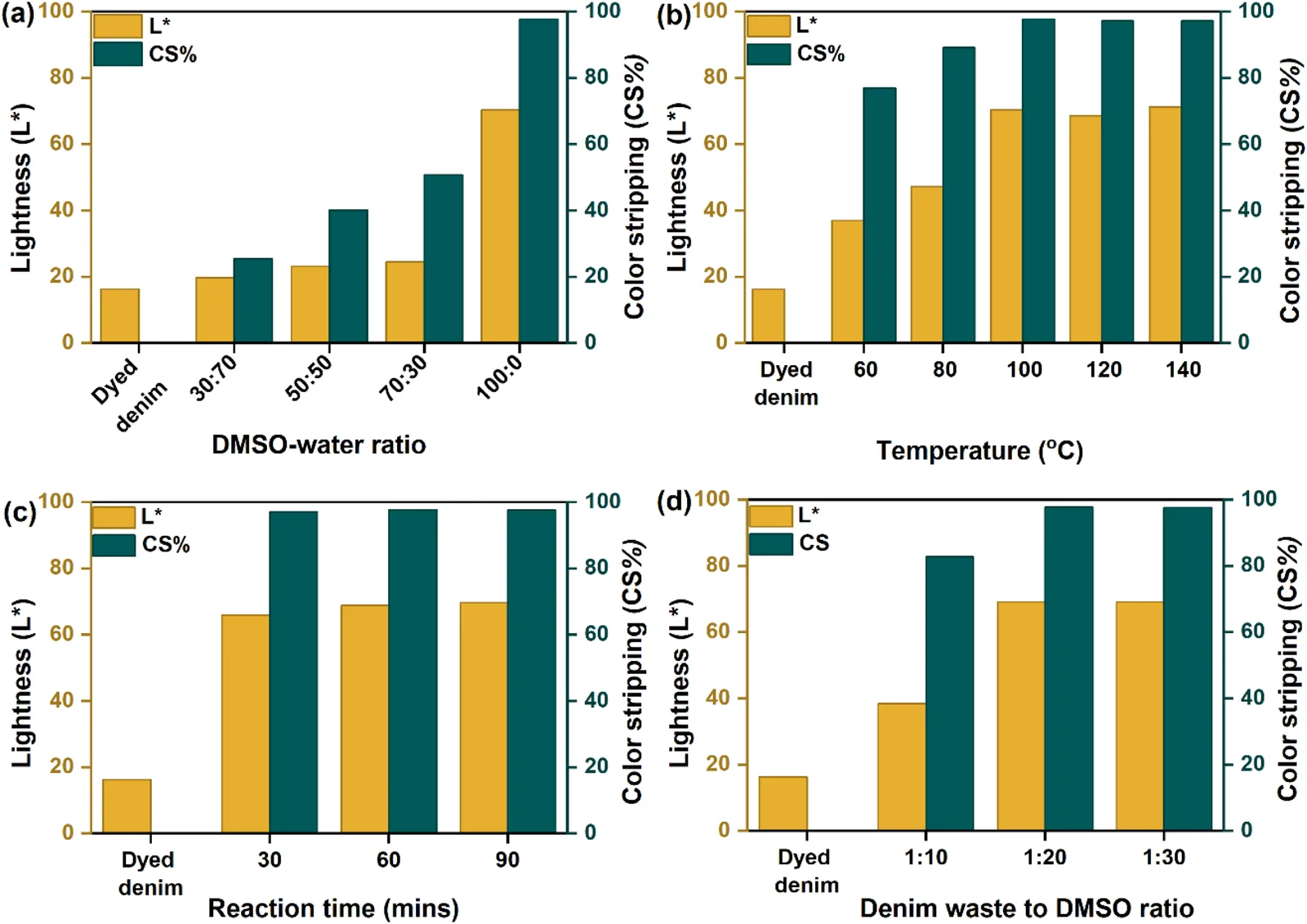
Solvent tuning and strategic process optimization of a DMSO-based color stripping for indigo-dyed denim textile waste. DMSO-water tuning at 100 °C for 60 min, maintaining M : L ratio of 1 : 20 (a); solvent-based process optimization by varying the temperature (b), reaction time (c), and material-to-liquor ratio (d).

The color stripping process was first tuned with DMSO and then optimized other process parameters such as temperature, treatment time, and material to liquor ratio (Fig. 1). The goal was to maximize the indigo dye removal and preserve the strength of the cellulose substrate. The DMSO: water composition showed a decisive influence on the color stripping performance. Color stripping increased with increasing DMSO content, and pure DMSO (100%) provided the highest stripping efficiency (Fig. 1a). This behavior is likely due to the high polarity and aprotic nature of DMSO, which enables strong interactions with the π-conjugated structure of indigo and enhances its solubility.^27^ Increasing the water content reduces the effectiveness of solvation of the medium and limits dye extraction from dyed denim due to decreased dye solubility. Based on these findings, pure DMSO was selected for the subsequent process optimization.

Color stripping increased as the temperature increased up to 100 °C. Above 100 °C, the stripping efficiency reached a plateau and did not improve significantly (Fig. 1b). The improved performance at higher temperatures is due to the dye solubility in DMSO and faster diffusion of the solvent into the fibers. Higher temperatures also weaken the interactions between indigo dye and cellulose and facilitate dye desorption. Since no significant improvement was observed beyond 100 °C, it was selected as the optimal temperature.

Stripping efficiency increased rapidly at the beginning and gradually reached saturation (Fig. 1c). This may have happened due to the desorption surface bound indigo dye and extraction from the outer surface where DMSO penetration is easy. Once the readily accessible dye is removed, further treatment yields only marginal improvement. Moreover, the 60 min treatment resulted in more uniform color stripping compared to the 30 min treatment.

Color stripping increased with increasing M : L ratio due to a high amount of DMSO available per unit mass of denim fabric, especially when moving from low to moderate values (Fig. 1d). For a practical perspective, an M : L ratio of 1 : 20 could be an effective balance between stripping efficiency and DMSO economy. To further evaluate color stripping performance under solvent-rich conditions, experiments were conducted at an elevated M : L ratio of 1 : 60 at 100 °C using fresh DMSO for various treatment time intervals (Fig. S4). However, the M : L ratio of 1 : 20 remains logical with optimized time and temperature.

**Fig. 2:**
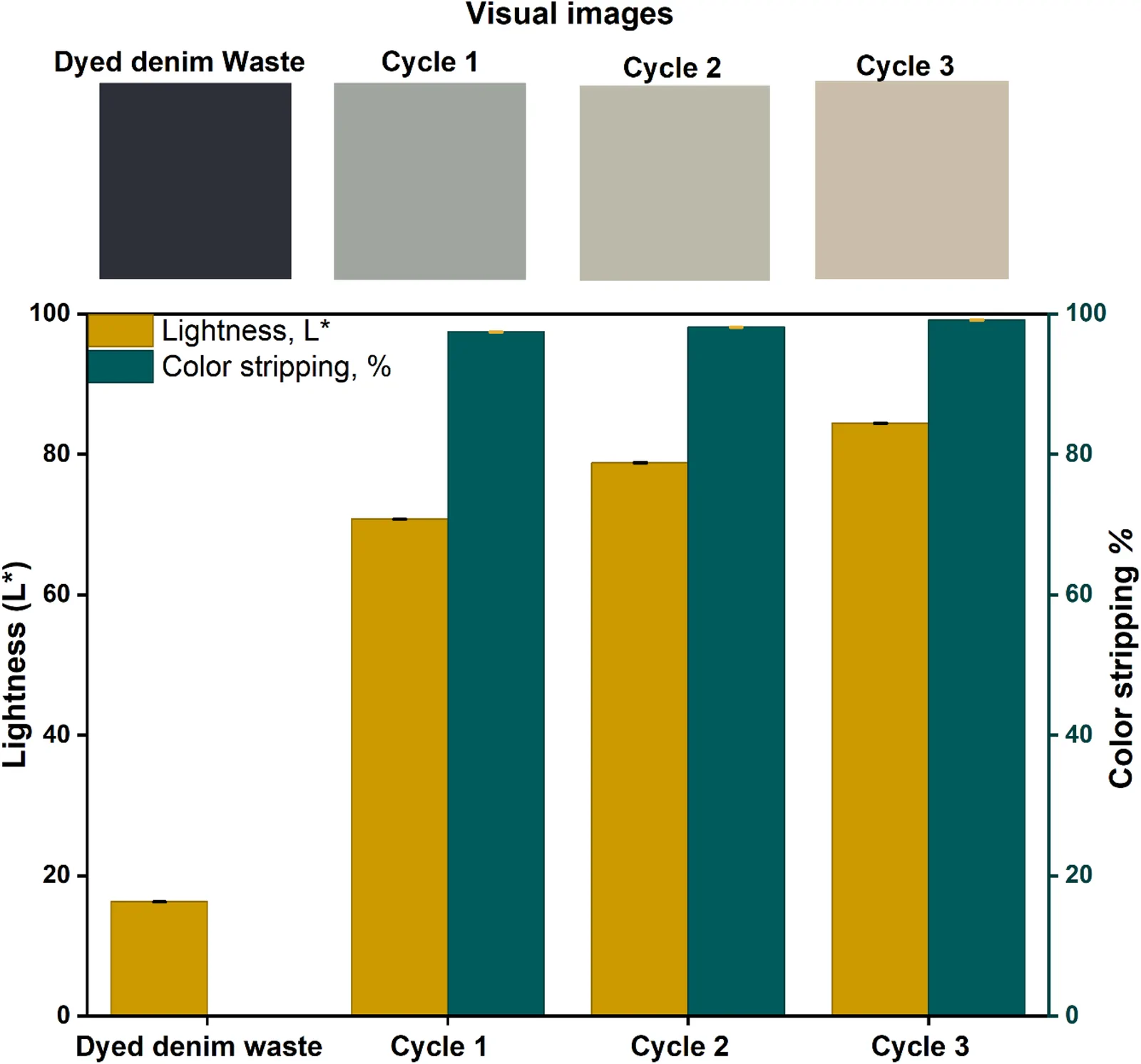
Relationship between color stripping cycles and stripping performance (each treatment cycle was conducted at a material-to-liquid ratio of 1 : 20 for 60 min at 100 °C, using fresh DMSO in every cycle).

The first color stripping cycle was carried out at optimized process conditions (100 °C for 60 min with an M : L ratio of 1 : 20) using fresh DMSO. This treatment removed more than 97% dyes from denim waste. However, a small amount of residual dye remained, and the fabric was still colorful with a lightness of about 70 (Fig. 2). Therefore, additional treatment was required to obtain 100% color removal and a white appearance. The subsequent 3 cycles are sufficient, and they showed more than 99% color stripping with lightness values above 84. Mainly, the residual dye was removed that was located in less accessible areas of the yarn and fabric structure during successive stripping.

The color stripping of indigo from dyed denim using DMSO is primarily governed by a physical extraction mechanism rather than chemical degradation. Although indigo does not form covalent bonds with cellulose, it is strongly retained within the fiber matrix through a combination of intermolecular interactions, including hydrogen bonding, hydrophobic interactions, π–π stacking between aromatic structures, and van der Waals forces. These interactions, along with physical entrapment within the amorphous regions of cellulose, contribute to the high dye–fiber affinity and resistance to removal by aqueous systems.

DMSO, a highly polar aprotic solvent with strong solvating ability, penetrates into the amorphous regions of cellulose and disrupts the intermolecular interactions. Its ability to solvate π-conjugated systems enhances the dissolution of indigo molecules, while fiber swelling increases chain mobility and facilitates the release of entrapped dye. This combined effect enables efficient extraction of indigo without breaking the cellulose backbone, thereby preserving the structural and mechanical integrity of the fiber. The process is therefore driven by solvent-assisted desorption and diffusion rather than chemical modification of either the dye or the polymer.

### Performance analysis of recovered DMSO

**Fig. 3:**
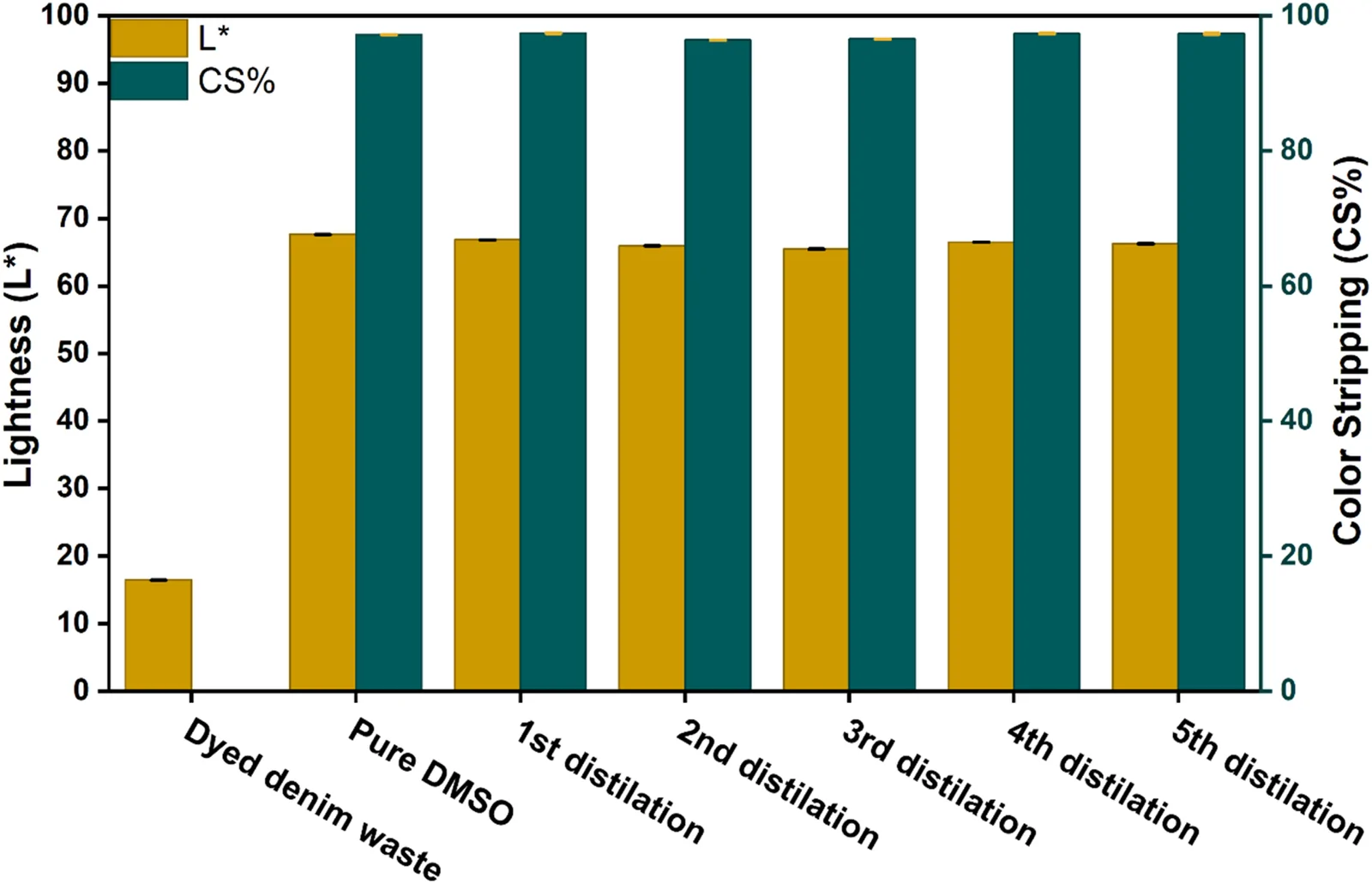
Color stripping performance with recovered DMSO through successive distillation cycles (1st to 5th).

Vacuum distillation was used for the recovery of solvent and separate extracted indigo. The performance of recovered DMSO was evaluated by reusing it for color stripping of dyed denim waste (Fig. 3). For each cycle, the DMSO from the previous stripping step was distilled, and the distillate was reused. This experiment directly assessed the feasibility of a closed-loop solvent system.

Color stripping efficiency remained high (above 96–98%) for pure and distilled DMSO at least up to five distillation cycles. No noticeable decrease in performance was observed. In addition, the lightness values of all cycles are identical with recovered DMSO. This may have happened due to no relevant solvent degradation occurring under the applied stripping and vacuum distillation conditions.

### Purity analysis of recovered indigo dye and DMSO

The DMSO recovery efficiency achieved after vacuum distillation was 99.4%. The total energy consumption of the distillation process was 0.08934 kWh, of which 0.048393 kWh was consumed by the stirrer and heating system, and 0.040948 kWh by the vacuum system. The solvent loss during one cycle was 1.8%, comprising 1.2% loss during the color stripping process, and 0.6% loss during the distillation step.

Fig. S5 shows the FTIR spectrums of indigo dye recovered after DMSO-based color stripping followed by distillation, commercial indigo powder, and undyed cotton fabric. As the recovered dye may contain trace amounts of cotton fibers and residual DMSO following solvent evaporation, the resulting FTIR spectrum is expected to comprise overlapping bands originating from indigo, cellulose, and DMSO.

In the high-wavenumber region, a broad band near 3430–3270 cm^−1^ corresponds to N–H stretching and the chelated C=O⋯H–N hydrogen-bonded structure of indigo.^28^ This band appears broader than expected for pure indigo, it may overlap with O–H stretching from residual cotton cellulose.^29^

In the mid-wavenumber region, a strong band between 1700 and 1580 cm^−1^ is assigned to the conjugated C=O, C=C, and N–H system of the indigo chromophore, confirming that the core dye structure survived the stripping and distillation process. The aromatic ring stretching bands near 1480 and 1455 cm^−1^ further confirm the presence of intact indigo. In the lower-wavenumber fingerprint region, any peak near 1050–1040 cm^−1^ is attributed to the S=O stretching of DMSO, indicating that some solvent remains in the sample.^30^ A peak near 1030–1160 cm^−1^ may instead, or additionally, arise from C–O and C–O–C stretching in cellulose, pointing to entrained cotton fiber particles. Finally, the low-frequency bands near 700–698 cm^−1^, assigned to C=O wagging, serve as an additional fingerprint confirming the identity of indigo in the recovered material.

The distilled liquid material was evaluated by ^1^H NMR spectroscopy. Small aliquots were analyzed to assess purity and residual water. The spectrum (Fig. S6) shows a distinct signal at *δ* 2.54 ppm assigned to DMSO and a minor signal at *δ* 3.32–3.35 ppm attributable to water, consistent with the chemical shift reported by Gottlieb *et al.*^31^ Integration (Table S1) indicates water contents of 5% (initial), 8% (after one cycle), and 12% (after five cycles). Drying of dimethyl sulfoxide (DMSO) can be achieved using activated molecular sieves or calcium hydride (CaH_2_).^32^ Further drying of the DMSO was not pursued. Only baseline noise was observed beyond these signals; no additional significant peaks were detected, and no DMSO-soluble impurities were found relative to the initial sample. Notably, the baseline noise decreased after five cycles.

Results indicate the presence of residual cotton fibers and DMSO in the recovered dye. Results also show that DMSO can effectively strip and recover indigo dye from denim fabric, while preserving its chemical integrity. However, the distillation and purification steps require further optimization to reduce solvent and fiber impurities. Removing these impurities is important for the reuse of recovered indigo in future dyeing applications within a circular textile framework.

### Mechanical behaviour of denim waste

**Fig. 4:**
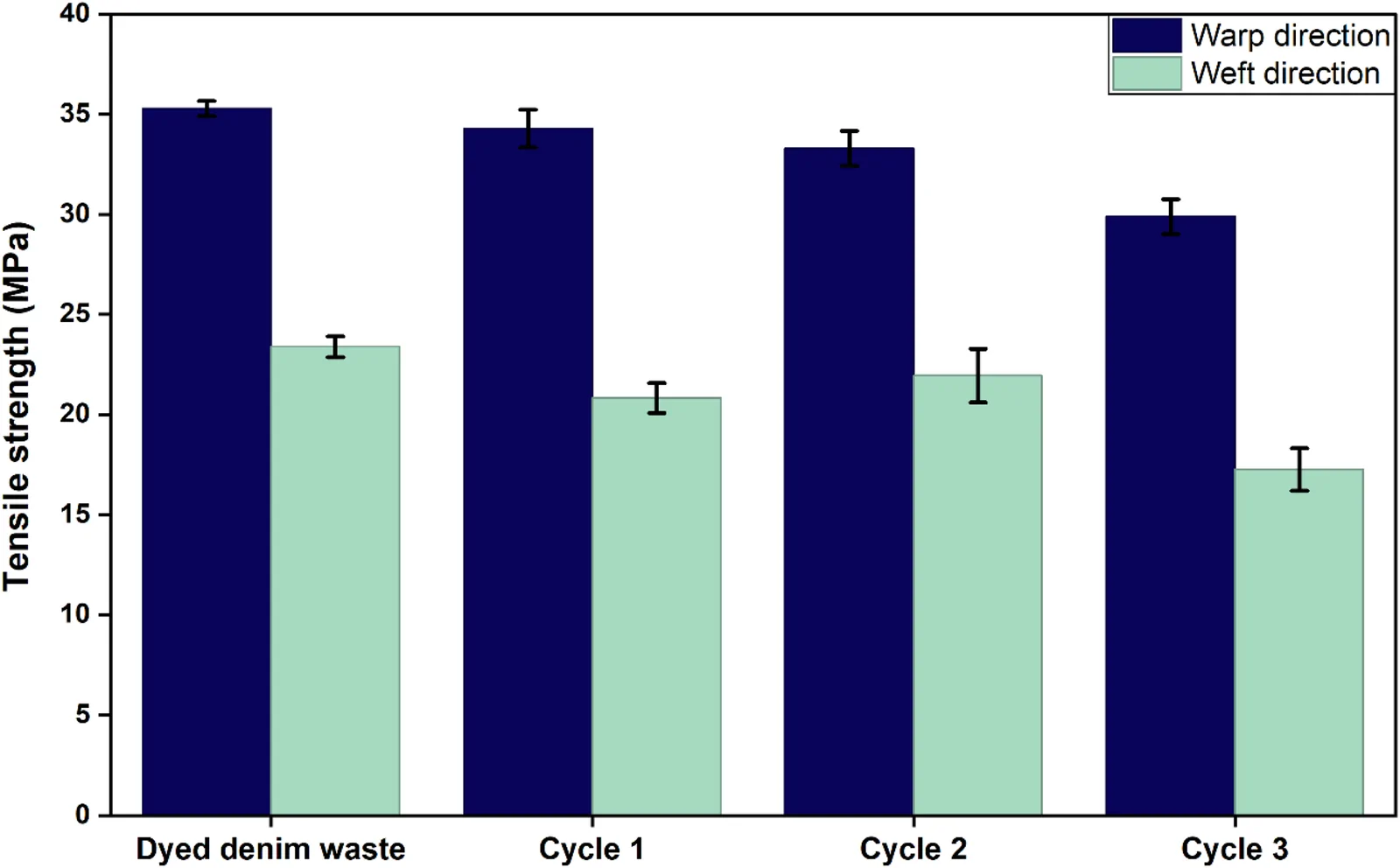
Changes of tensile strength in warp and weft direction during color stripping with DMSO.

The tensile strength of color-stripped denim waste was evaluated, and the results are presented in Fig. 4. The tensile strength retention was found to be >98% in both warp and weft directions after the first stripping cycle under optimized conditions. A further reduction in tensile strength was observed with additional stripping cycles. However, the total strength loss remained below 6% up to the third stripping cycles. DMSO is well-known to swell cellulose by penetrating the amorphous regions of the polymer matrix.^33^ Due to its strong polarity and high solvating ability, DMSO can disrupt intermolecular hydrogen bonding within cellulose, increasing chain mobility and free volume. This swelling effect may alter the orientation and packing of cellulose chains, particularly in the amorphous domains where molecular organization is less ordered compared to crystalline regions.^34^ Such structural modifications can influence the mechanical properties of the fibers. Changes in chain alignment and intermolecular interactions may reduce intermolecular cohesion, potentially leading to a decrease in tensile strength. The extent of this effect depends on factors such as solvent exposure time, temperature, concentration, and the degree of swelling. Therefore, while DMSO can enhance dye removal by improving solvent penetration and disrupting dye–fiber interactions, it may also induce structural changes in cellulose that need to be carefully controlled to preserve fiber integrity. The slight strength loss of denim waste may be caused by repeated stresses such as wetting, swelling, drying, and friction in handling, rather than by solvent induced degradation of the polymer chain.

### Degree of polymerization (DP) and intrinsic viscosity (IV)

**Fig. 5:**
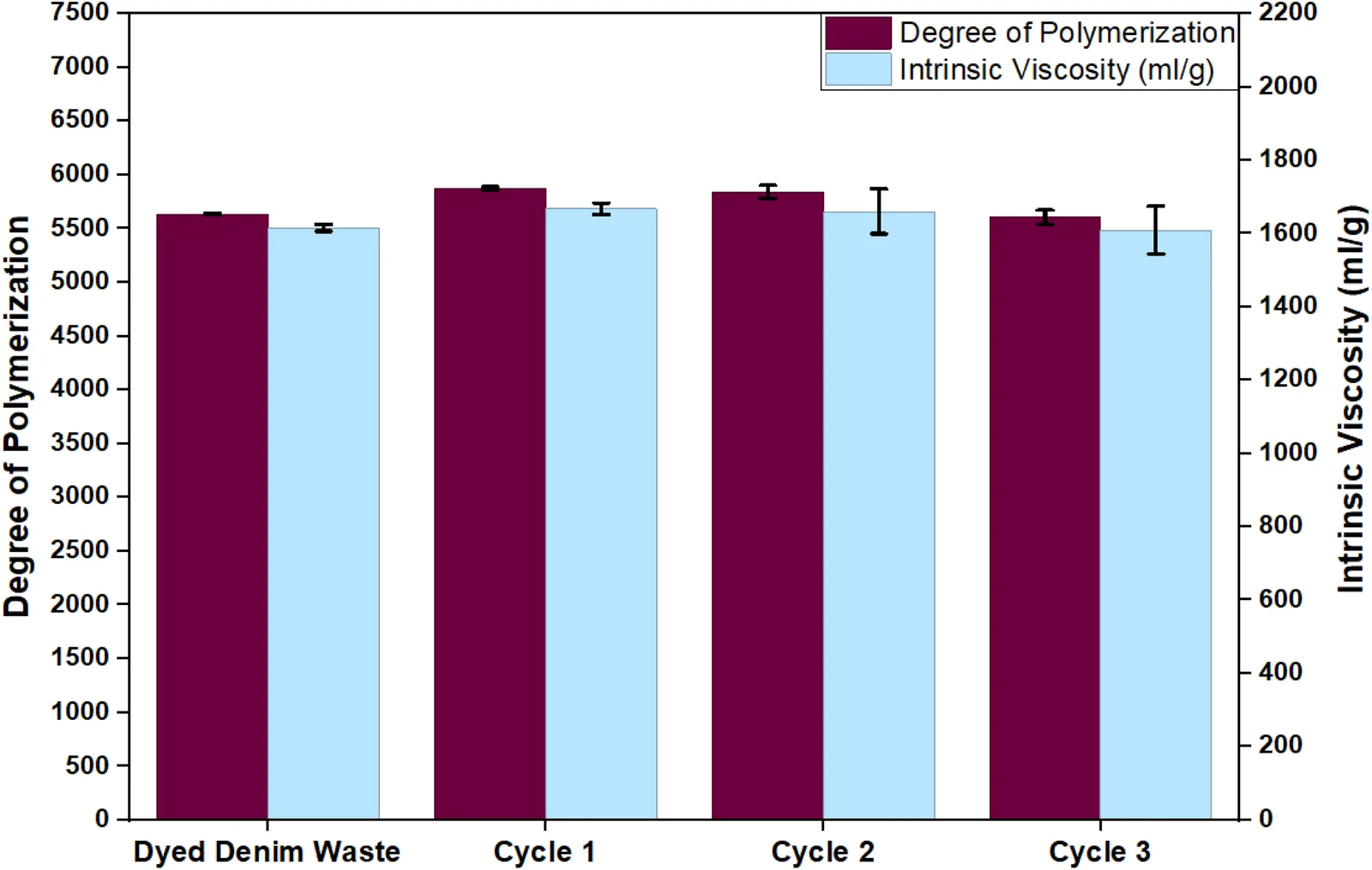
The effect of DMSO-based color stripping on the degree of polymerization and intrinsic viscosity of the fabrics in different cycles.

Fig. 5 illustrates the changes in degree of polymerization and intrinsic viscosity of dyed denim waste after successive stripping cycles. Overall, the intrinsic viscosity values measured after 1–3 cycles are similar within experimental error and the possible lower viscosity of the unstripped sample is affected by the presence of the dye that does not contribute to the viscosity. The solvent primarily attacks the surface-bound dye molecules, which are mostly located in the amorphous region of the cellulosic structure, thus leaving the *β*-1,4-glycosidic bonds of cellulose.^34^ The stripping mechanism is mainly focused on disrupting dye–fiber interaction, rather than degrading overall cellulosic structure. Indigo dyes in denim are mainly entrapped or held within the structure, which can be cleaved without dismantling the cellulose network. Additionally, the strong hydrogen bonding network in the crystalline region restricts solvent diffusion, thereby remaining intact. Thus, unlike harsh chemical treatment,^15^ DMSO-assisted stripping prevents excessive chain scissions. As a result, entrapped indigo dyes are removed without affecting the backbone of the cellulosic structure, maintaining its structural performance even after successive cycles.

### Surface morphology analysis

**Fig. 6:**
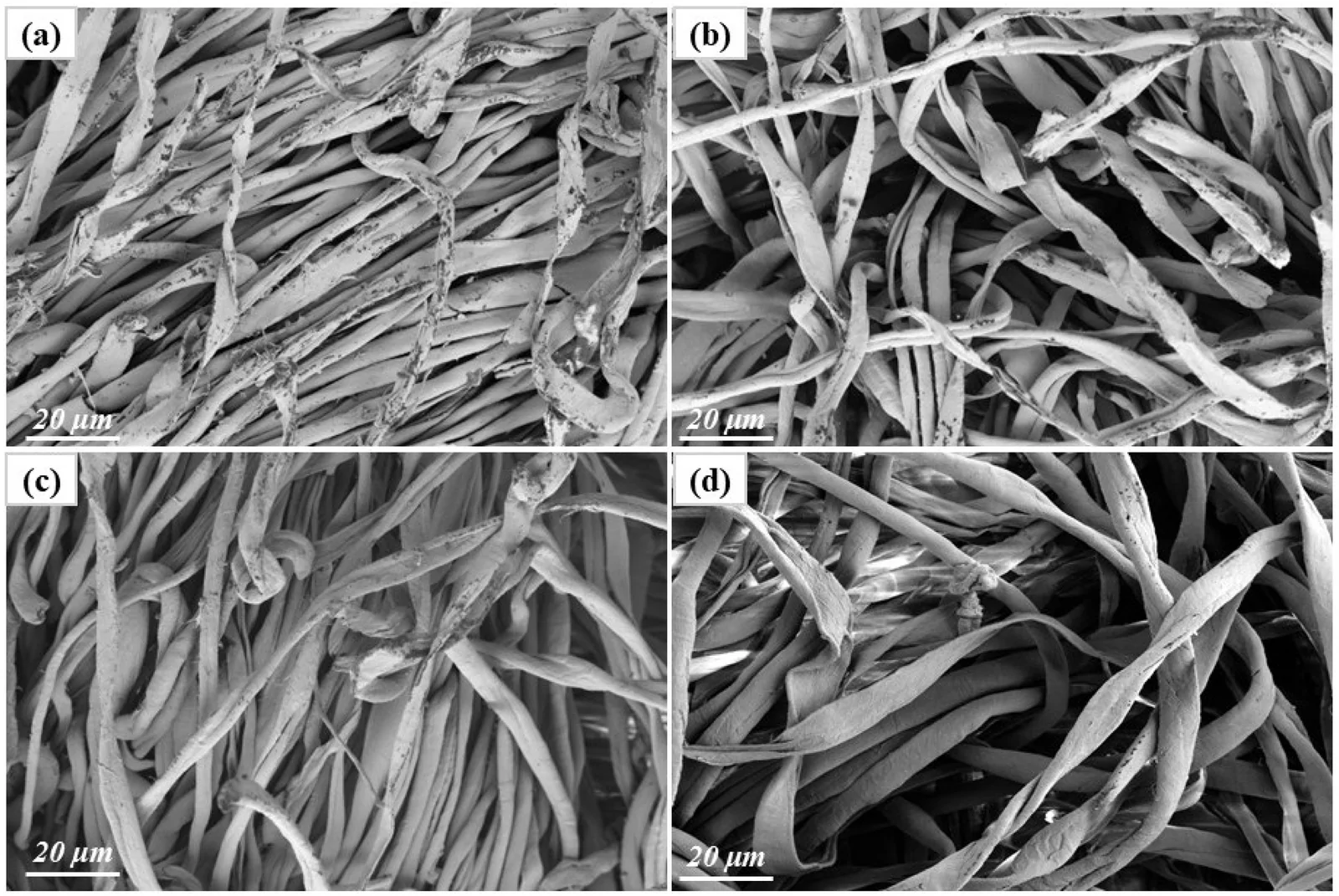
SEM images of indigo dyed denim fabric (a) and solvent-based color-stripped fabric after 1st cycle (b), 2nd cycle (c), and 3rd cycle (d) at 250× magnification.

The effect of DMSO based color stripping process on the fabric microstructure was identified by SEM, and corresponding images are shown in Fig. 6. The dyed denim waste shows a relatively smooth fiber surface, covered by indigo dye particles and deposits on and between fibers (Fig. 6a). No major damage, cracks, or fibrillation is visible, confirming that the dyed denim is structurally intact. After the first stripping cycle, the yarn architecture remains unchanged (Fig. 6b). The main evident changes are a clear reduction of indigo dye particles. This observation is in line with the high color stripping efficiency achieved under optimized conditions (Fig. 1). No microcracks and surface damage are observed, which indicates that DMSO selectively extracts the dye from the denim surface without attacking cellulose or yarn structure. After the second (Fig. 6c) and third cycles (Fig. 6d), the denim fabric still shows well defined pattern. The fiber surface appears cleaner due to further removal of residual dye.

### Elemental analysis

The elemental compositions of dyed denim waste and stripped denim fabrics are tabulated in Table 1. The significant change is observed in the nitrogen content. Dyed denim waste contains 0.40% nitrogen. After stripping, the nitrogen content decreases to 0.14% in the first cycle. Further, the nitrogen content reduction was found to be 0.12 and 0.11% in the second and third cycle respectively. Since denim fabric is not essentially nitrogen-free, the nitrogen originates from indigo dye as well as the cellulose matrix. The residual nitrogen value of 0.11% after the third cycle may represent the natural value in cotton.^35^ The reduction from 0.40% to 0.11% corresponds to about 2.7% decrease in nitrogen content. This trend is in line with the high color stripping efficiencies measured spectrophotometrically (Fig. 1; eqn (2)).

**Table 1:** Elemental composition of dyed and color-stripped denim fabrics

Sample type	Elements (%)			
	Carbon	Hydrogen	Nitrogen	Sulfur
Dyed denim	43.19 ± 0.04	6.00 ± 0.13	0.40 ± 0.01	0.00
Stripped denim (cycle 1)	42.76 ± 0.31	6.07 ± 0.07	0.14 ± 0.01	0.00
Stripped denim (cycle 2)	42.35 ± 0.19	6.10 ± 0.03	0.12 ± 0.03	0.00
Stripped denim (cycle 3)	42.01 ± 0.19	5.75 ± 0.22	0.11 ± 0.02	0.00

There is no significant change in the C/H ratio, indicating that the cellulose backbone remains unaffected and no additional heteroatoms are incorporated during the process. This observation supports the preservation of the cellulose structure in the denim fabrics. Moreover, sulfur was not detected (0.00% within detection limits) in either the dyed or stripped samples. This suggests that no sulfur-containing auxiliaries are present in the denim waste, and that the DMSO-based stripping and subsequent washing steps do not leave detectable sulfur residues on the fabric, despite DMSO containing sulfur in its molecular structure.

### Structural behaviour analysis

**Fig. 7:**
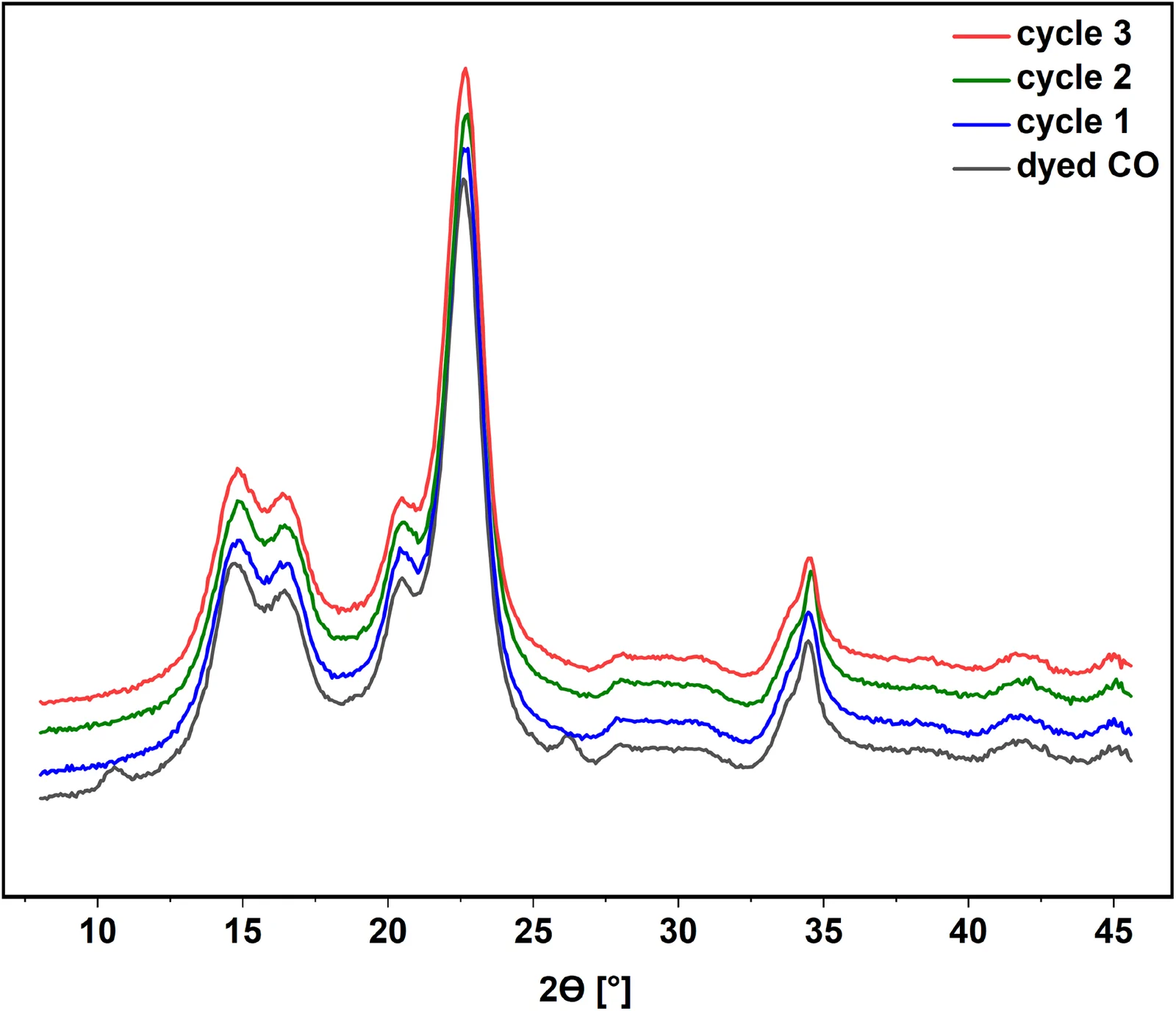
X-ray diffraction patterns of indigo dyed denim waste and DMSO-based color-stripped denim fabrics during multiple treatment processes.

XRD analysis shows that the DMSO-based color-stripping process does not change the crystallinity and/or arrangement of the polymer of cellulose (Fig. 7). The main diffraction peaks (110), (110) and (200) of cellulose I and the crystallinity index of 47% remain essentially the same.^36^ The reached crystallinity index is close to the values reported within earlier works (41–44%).^37^ This result suggests that the structural order of the cellulose phase is preserved even after multiple treatment cycles (Table S2). However, in the dyed denim waste (dyed CO), indigo shows 2 prominent reflexes at ∼11 and 26° (2*θ*).^18,38^ At the same time as the dye is removed, the indigo peaks disappear.

### Thermal behaviour analysis

**Fig. 8:**
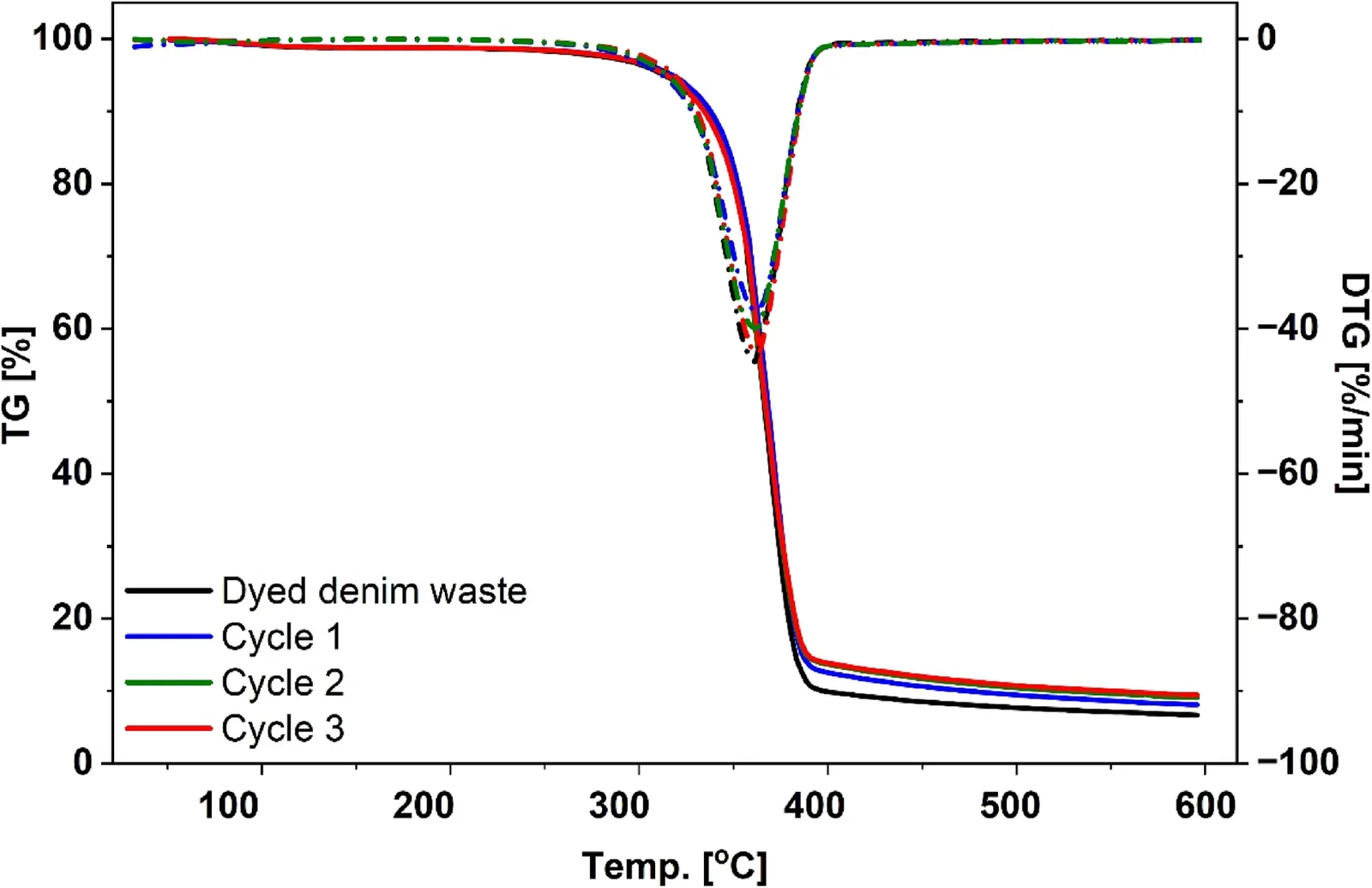
TGA curves of indigo dyed denim and DMSO-based color-stripped denim fabrics during multiple treatment processes.

The thermogravimetric degradation curves of the indigo-dyed and DMSO-treated color-stripped denim fabrics are presented in Fig. 8, and the corresponding thermal degradation parameters are summarized in Table S3. All samples exhibit a minor mass loss below 100 °C, which is attributed to the evaporation of physically adsorbed moisture and trapped volatiles within the fibre structure.^39,40^ The main degradation stage occurs in the range of approximately 340 to 390 °C and corresponds to the pyrolytic decomposition of cellulose, involving depolymerization, dehydration, and cleavage of glycosidic linkages of the anhydroglucose units, ultimately leading to char formation.^41^*T*_onset_ (343 to 346 °C), *T*_max_ (366 to 367 °C), and *T*_endset_ (383 to 387 °C) values are nearly identical for the dyed and stripped samples, indicating that repeated DMSO-based stripping cycles do not significantly affect the intrinsic thermal stability of the cellulose polymer chain.^42^ Indigo dyes, within the fibre, primarily through physical entrapment and hydrogen bonding rather than covalent bonding,^43^ may slightly influence the initial degradation behaviour and char yield. The marginal differences in residual mass at 600 °C (7 to 9%) can therefore be associated with the contribution of indigo's aromatic structure to char formation during pyrolysis.^44^

### Redyeability analysis of color-stripped denim

**Fig. 9:**
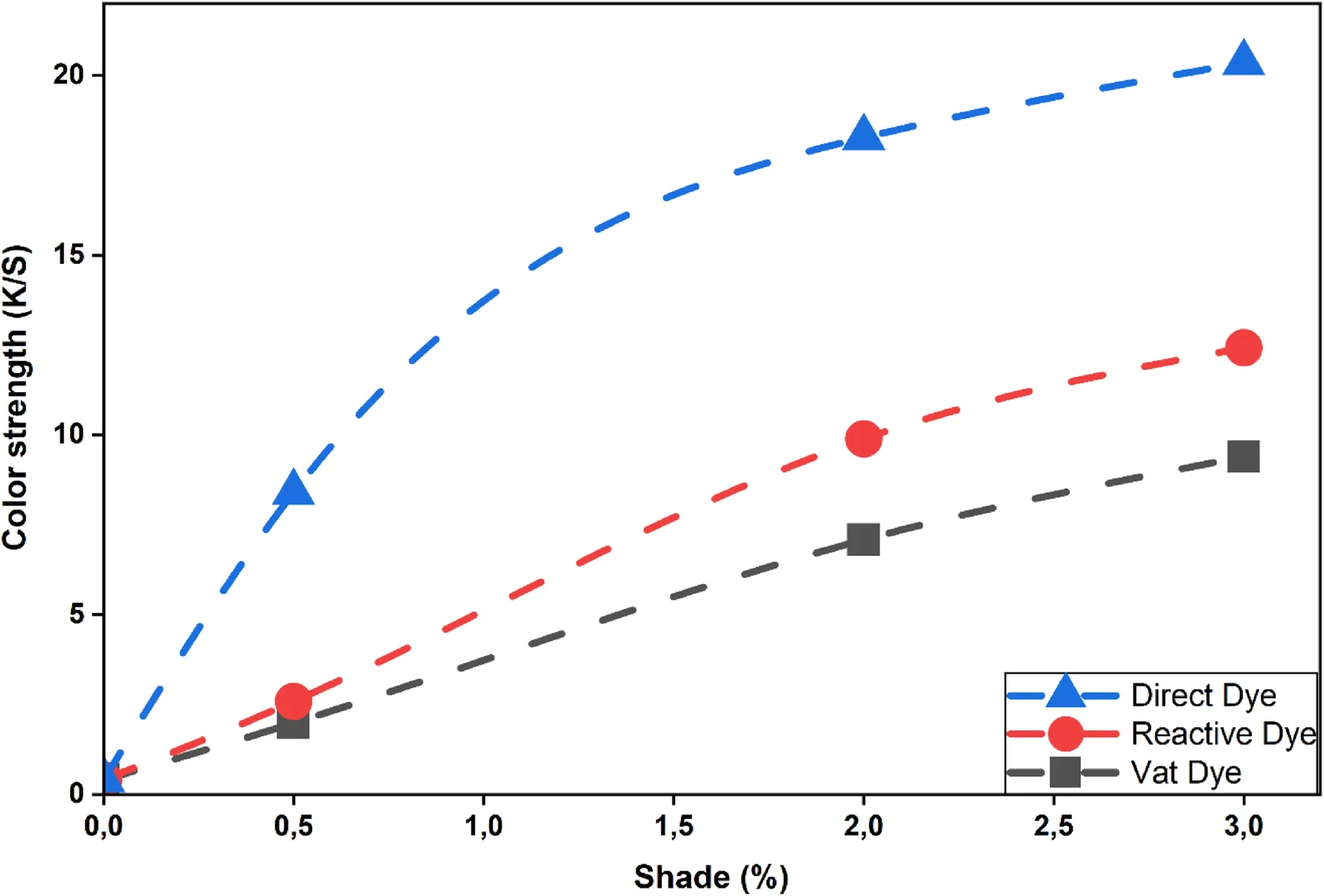
Color strength performance pattern of DMSO-based color-stripped denim fabrics with different commercial dyes such as Bezathren Blue RS (Vat dye), Bezaktiv Red HP-BL (Reactive dye) and Tubantin Orange GGLN 200 (Direct dye) and various shade percentages.

The re-dyeability of the color-stripped denim fabrics was evaluated with three commercial dye types: Bezathren Blue RS (Vat dye), Bezaktiv Red HP-BL (Reactive dye) and Tubantin Orange GGLN 200 (Direct dye) at different shade levels (0.5, 2.0, and 3.0% o.w.f.). Color strength, fixation efficiency, ISO brightness, color fastness, and levelness were analyzed, and results are presented accordingly in Fig. 9 and Table 2 and 3. Moreover, the CIE L*a*b* color space of redyed color-stripped denim is tabulated in Table S3 and the suggested interpretation of Δ*E*_cmc_ values in Table S5.

**Table 2:** Dyeability characteristics of DMSO-based color-stripped denim fabrics: fixation efficiency, ISO brightness, wash and rubbing fastness, and redyed fabric images for different commercial dyes and shade percentage

Dye type	Shade, %	*F*_eff_%	ISO brightness	Wash fastness		Rubbing fastness		Image
				C.C.	C.S.	Dry	Wet	
Vat dye	0.5	90.89	36.24	4–5	4–5	4–5	4–5	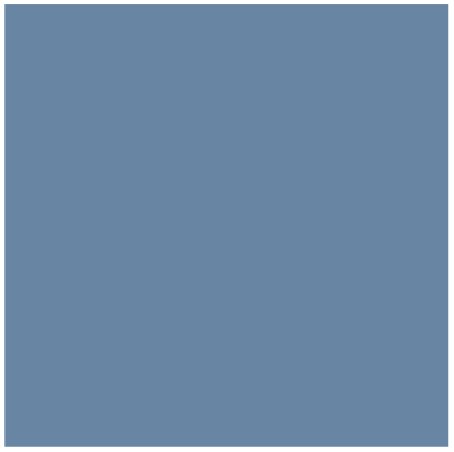
	2.0	68.59	21.62	4–5	4	4–5	3–4	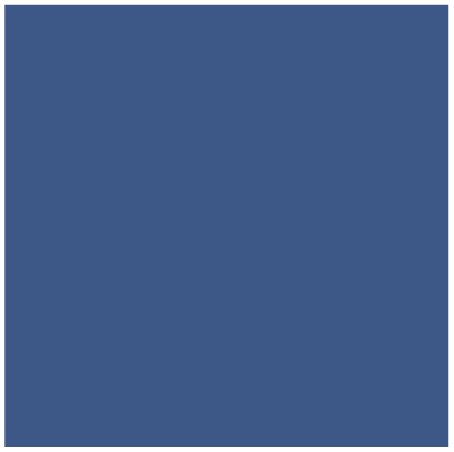
	3.0	63.13	17.11	4–5	3–4	4–5	3–4	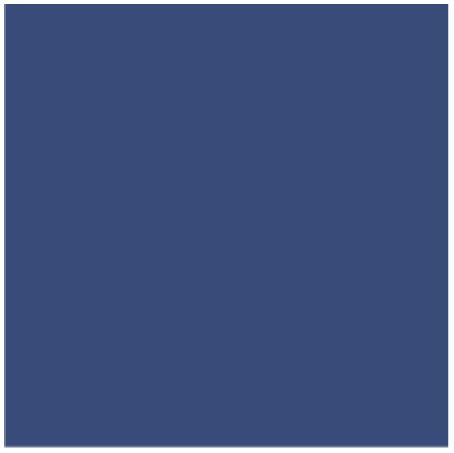
Reactive dye	0.5	90.34	27.36	5	5	5	4–5	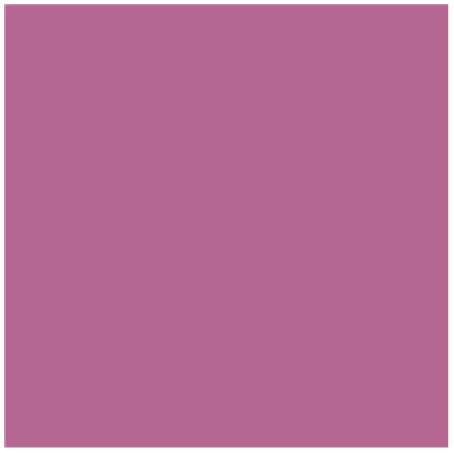
	2.0	84.46	13.11	5	4–5	5	4–5	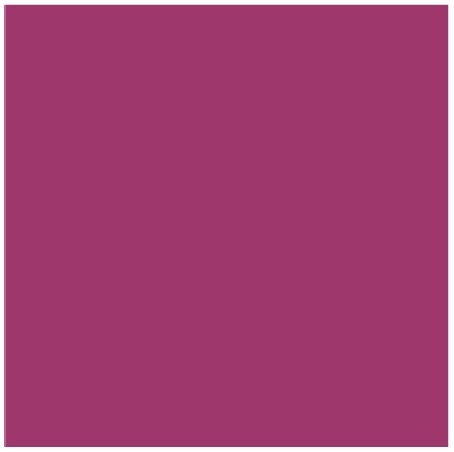
	3.0	89.12	11.03	5	4–5	5	4–5	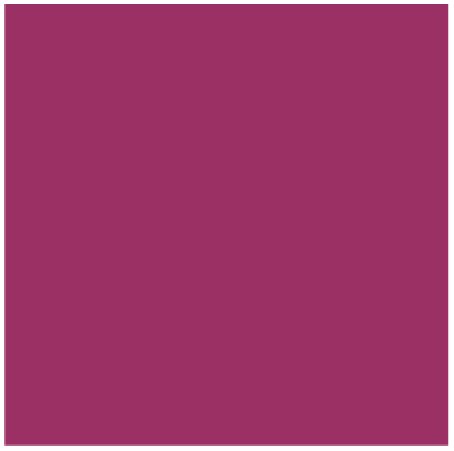
Direct dye	0.5	79.24	5.82	4	4	4–5	4	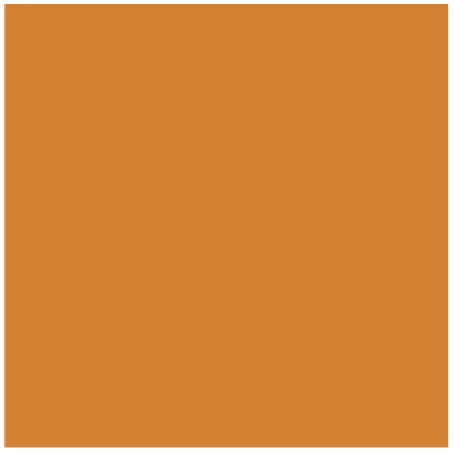
	2.0	78.13	2.80	4	3–4	4–5	3–4	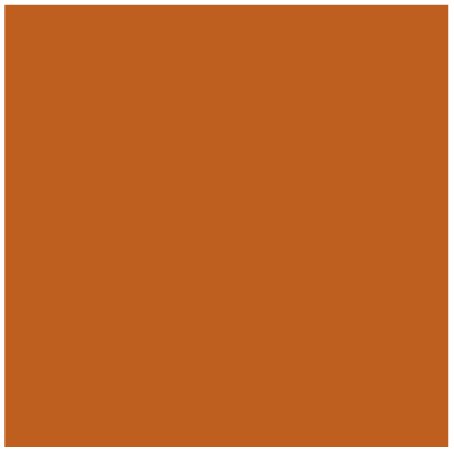
	3.0	74.11	2.52	4	3	4–5	3–4	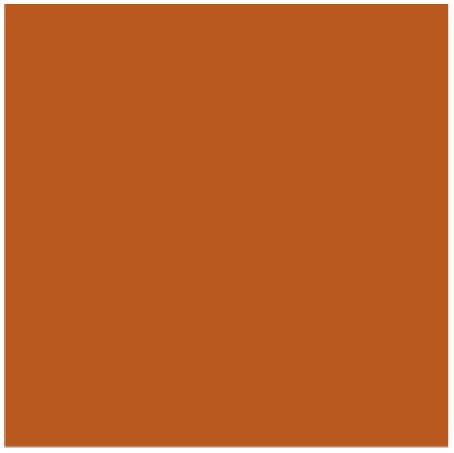

**Table 3:** Color levelness (Δ*E*_cmc_) of re-dyed denim fabrics with different commercial dyes (Bezathren Blue RS, Bezaktiv Red HP-BL, and Tubantin Orange GGLN 200) and shade percentage

Dye type	Shade, %	Δ*E*_cmc_ of re-dyed denim fabric										Average Δ*E*_cmc_
		Batch readings										
		R-1	R-2	R-3	R-4	R-5	R-6	R-7	R-8	R-9	R-10	
Vat dye	0.5	Standard	0.39	0.12	0.52	0.87	0.72	0.47	0.29	0.4	0.37	0.46
	2.0		0.89	0.91	0.08	0.76	0.86	0.89	0.59	0.19	0.47	0.63
	3.0		0.62	0.36	0.39	0.47	0.61	0.56	0.35	0.93	0.57	0.54
Reactive dye	0.5		0.24	0.32	0.27	0.55	0.16	0.26	0.46	0.64	0.60	0.39
	2.0		0.29	0.09	0.28	0.23	0.35	0.32	0.19	0.21	0.27	0.25
	3.0		0.39	0.7	0.17	0.2	0.23	0.37	0.25	0.61	0.51	0.38
Direct dye	0.5		0.24	0.36	0.08	0.19	0.45	0.3	0.11	0.38	0.18	0.26
	2.0		0.11	0.27	0.23	0.59	0.68	0.46	0.26	0.28	0.30	0.35
	3.0		0.31	0.43	0.24	0.54	0.57	0.66	0.56	0.41	0.55	0.47

The color strength (*K*/*S*) increased consistently with increasing dye concentration from 0.5 to 3% shade for all three dyes (Fig. 9), with no evidence of early saturation. Even at the highest shade level (3%), the *K*/*S* values continued to increase, indicating that the DMSO treatment did not block or deactivate the dye-binding sites on the cotton fibers. This observation is consistent with the structural characterization, which showed that the fiber morphology and cellulose structure were largely preserved following the DMSO-based color stripping process. The cellulose crystallinity and fibre structure remain unchanged (Table S2), and elemental composition corresponds to clean cotton (Table 1). Hydroxyl groups in the cellulose structure are accessible and therefore vat, reactive, and direct dyes could be applied successfully to stripped denim fabric. Real images of re-dyed denim fabrics with different commercial dyes (Bezathren Blue RS, Bezaktiv Red HP-BL and Tubantin Orange GGLN 200) and various shade percentages are mentioned in Fig. S7.

The fixation efficiencies for Bezathren Blue RS (Vat dye), Bezaktiv Red HP-BL (Reactive dye) and Tubantin Orange GGLN 200 (Direct dye) dyes are found to be 73–94% which is considered consistently high across 0.5–3.0% shade. Some variations are observed as shade depth increases (Table 2). A small reduction in fixation efficiency for higher shade is expected because more dyes remain on the fiber surface and removed during washing. This behaviour is common for cotton dyeing and does not indicate substrate damage. The high fixation of reactive dye shows that hydroxyl groups in cellulose are formed covalent bond. The high performance of vat dye indicates that reduction and re-oxidation inside the fiber proceed normally. The good fixation of the direct dye confirms that hydrogen bonding and substantivity are maintained. ISO brightness decreases with increasing shade for all dyes (Table 2). Darker shades naturally show lower brightness values. The corresponding color co-ordinate (lightness, L*; redness, a*; blueness, b*; chroma, C*; and hue, h°) values of all redyed denims are presented in Table S4.

Redyed denim with Bezathren Blue RS shows very good to excellent fastness behavior (Table 2). After reduction and oxidation, vat dyes form insoluble pigments inside the fibers. This explains the very good resistance to washing and rubbing. On the other hand, Bezaktiv Red HP-BL shows excellent wash fastness (grade 5) and very good rubbing fastness.^45^ Reactive dyes form covalent bonds with cellulose. The results confirm that hydroxyl groups in cellulose remain fully reactive after DMSO stripping. Moreover, Tubantin Orange GGLN 200 also demonstrated good wash and rubbing fastness. The slightly lower staining fastness observed at higher shade depths is consistent with the non-covalent nature of direct dye fixation, which relies primarily on hydrogen bonding and other physical interactions with cellulose.

According to the color levelness interpretation scale, Δ*E*_cmc_ values of ≤0.20 indicate excellent levelness, values between 0.21 and 0.50 indicate good levelness, values between 0.51 and 1.00 indicate poor levelness, and values above 1.00 indicate very poor levelness (Table S5).^46^ The average Δ*E*_cmc_ values obtained for all dyes and shade depths ranged from 0.21 to 0.50, corresponding to good levelness. Furthermore, several measurements were close to or below 0.20, indicating excellent levelness and demonstrating the uniform dye uptake of the DMSO-treated denim.

## Conclusion

This study effectively develops a closed-loop, solvent-based strategy for the sustainable recycling of indigo-dyed denim waste. DMSO was used as a recyclable stripping medium, and it enabled highly efficient color removal and cellulose structural pattern. The color-stripped denim showed high lightness, which could facilitate subsequent re-dyeing and/or mixing with virgin cotton to prepare new textiles. The process integrates distillation-based recovery of both DMSO and indigo dye. Recovered DMSO was reused for at least 5 consecutive color stripping cycles without performance loss. By circumventing water-intensive and chemically harsh stripping practices, this approach advances textile processing consistent with circular economy principles. Some key findings of this approach are as follows:

• Neat DMSO outperforms aqueous DMSO systems and achieves ≥98% color stripping.

• Color stripped denim exhibits high lightness (L* >81) which could enable fibre-to-fiber recycling.

• Color-stripped denim showed excellent preservation of cellulose substrate with ≈98% tensile strength retention.

• Recovered DMSO showed excellent color stripping performance at least 5 times.

• Re-dyeing feasibility of stripped denim is supported by fastness and color levelness across vat, reactive, and direct dye types at varying shade depths.

Future work will focus on scaling up the process by developing continuous stripping-distillation systems with heat integration to reduce energy consumption. Further studies will also investigate the conversion of stripped fabrics into new fibers using Ioncell and Lyocell processes. Detailed techno-economic analysis and life cycle assessment will be carried out to compare environmental and economic performance with existing processes. In addition, extended cycling studies (beyond five cycles and, ideally, under continuous pilot-scale operation) will be conducted to confirm the long-term stability of both the solvent and the process. These studies will also evaluate the potential accumulation of trace impurities, water, and degradation products over repeated recovery and reuse cycles.

## Author contributions

Md. Reazuddin Repon: conceptualization, methodology, data collection, software, formal analysis, writing – original draft, writing – review and editing, visualization; Floriane Jacquin: methodology, formal analysis, investigation, data curation, writing – review and editing; Shubhajit Dutta: data collection, software, data curation, writing – original draft, formal analysis; Tonmoy Saha: data collection, software, formal analysis, data curation, writing – original draft; Inge Schlapp-Hackl: methodology, software, formal analysis, investigation, writing – original draft, writing – review and editing; Tapani Vuorinen: conceptualization, methodology, writing – review and editing, supervision and Ali R. Tehrani-Bagha: conceptualization, methodology, formal analysis, resources, writing – review and editing, supervision, project administration and funding acquisition.

## Conflicts of interest

The authors have no relevant conflicts of interests to disclose.

## Supplementary Material

RA-016-D6RA04250C-s001

## Data Availability

The data supporting the findings of this study are available within the article and its supplementary information (SI). Additional data related to this work are available from the corresponding author upon reasonable request. Supplementary information: seven figures (Fig. S1–S7) and five tables (Tables S1–S5) that provide supporting experimental procedures and characterization data. These include the color-stripping process, solvent and dye recovery, re-dyeing procedures, process optimization, FTIR and NMR analyses, and photographs of the re-dyed fabrics. The tables present NMR integrals and water content, crystallinity indices, TG/DTG thermal analysis results, CIE Lab* color coordinates, and Δ*E*_CMC_ color difference interpretation. See DOI: https://doi.org/10.1039/d6ra04250c.
