# Supplementary material for: Development of a closed-loop solvent-based recycling process for dyed denim waste
Source: RSC Adv. 2026 Jul 18;16(38):41498–510. doi: 10.1039/d6ra04250c (PMC13379290; doi:10.1039/d6ra04250c)
Supplement: Supplementary file 1 [file RA-016-D6RA04250C-s001.pdf]

Supplementary Information (SI) for RSC Advances.
This journal is © The Royal Society of Chemistry 2026

Supplementary Information

Development of a Closed-Loop Solvent-Based Recycling Process for Dyed Denim Waste

Md. Reazuddin Repon[1], Jacquin Floriane[1,2], Shubhajit Dutta[1], Tonmoy Saha[1], Inge Schlapp-Hackl[1], Tapani Vuorinen[1], and Ali Tehrani-Bagha[1,*]

[1] Department of Bioproducts and Biosystems, School of Chemical Engineering, Aalto University, Vuorimiehentie 1, 02150, Espoo, Finland

[2] Agro Toulouse, Institut National Polytechnique de Toulouse, Auzeville-Tolosane, France

Supplementary figures (S1-S7):

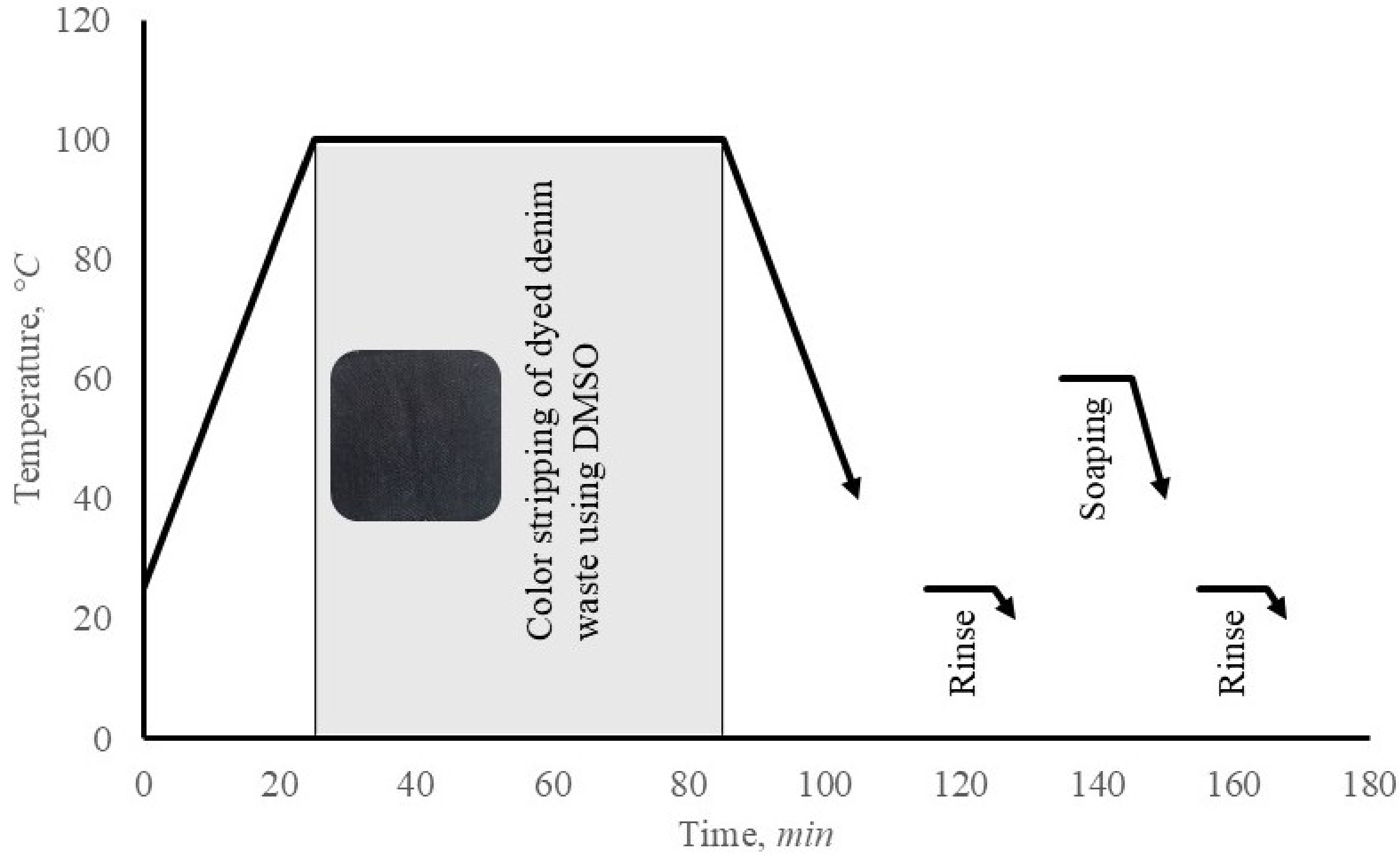

Figure S1. Process diagram of color stripping of indigo dyed denim waste using DMSO.

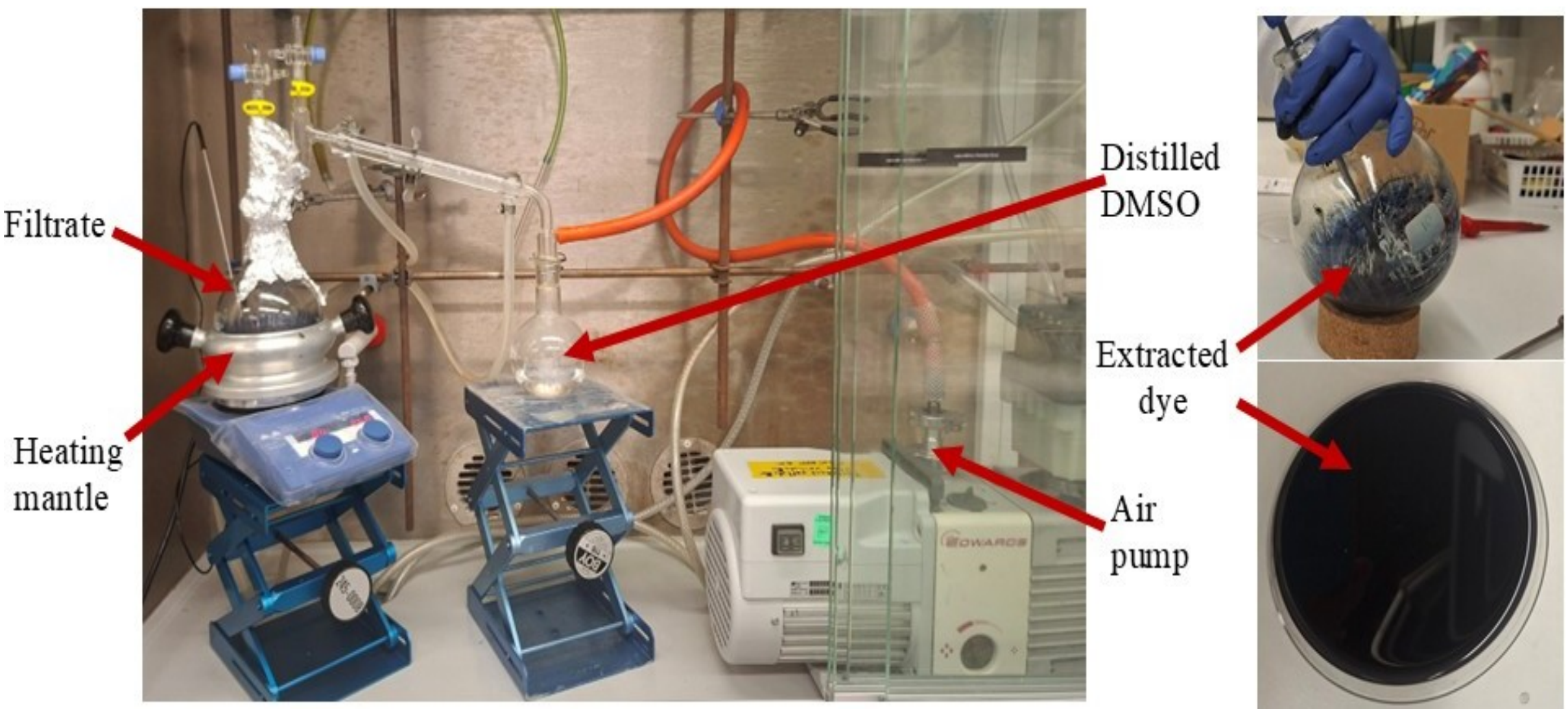

Figure S2. Solvent and dye recovery setup: distilled DMSO is a clear and indigo-free solvent (left side), and extracted indigo dye in petridish (right side).

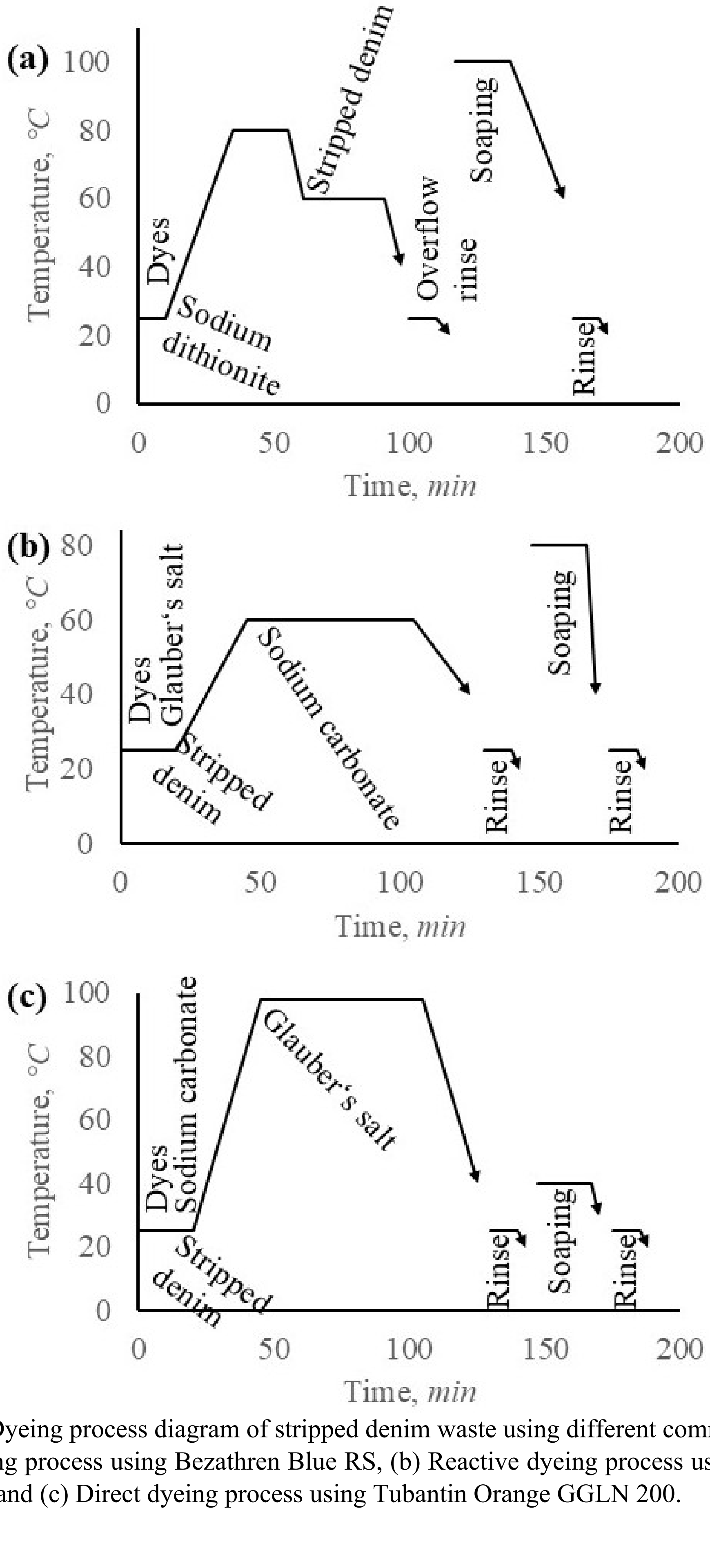

Figure S3. Dyeing process diagram of stripped denim waste using different commercial dyes: (a) Vat dyeing process using Bezathren Blue RS, (b) Reactive dyeing process using Bezaktiv Red HP-BL and (c) Direct dyeing process using Tubantin Orange GGLN 200.

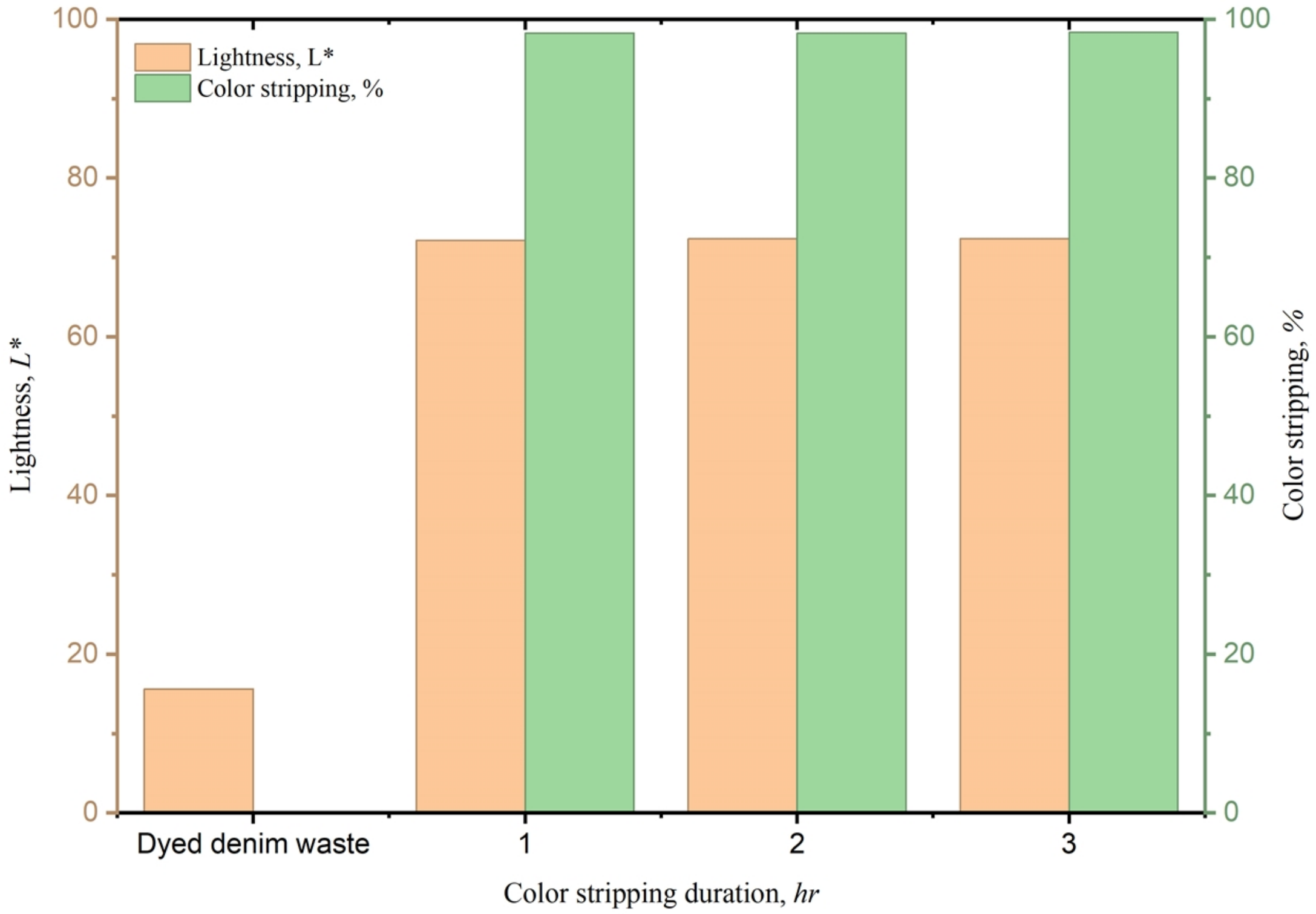

Figure S4. Relationship between color stripping duration and stripping performance at higher materials to liquor ratio (treatment was conducted at a material-to-liquid ratio of 1:60 for 60 min at 100 °C, using fresh DMSO)

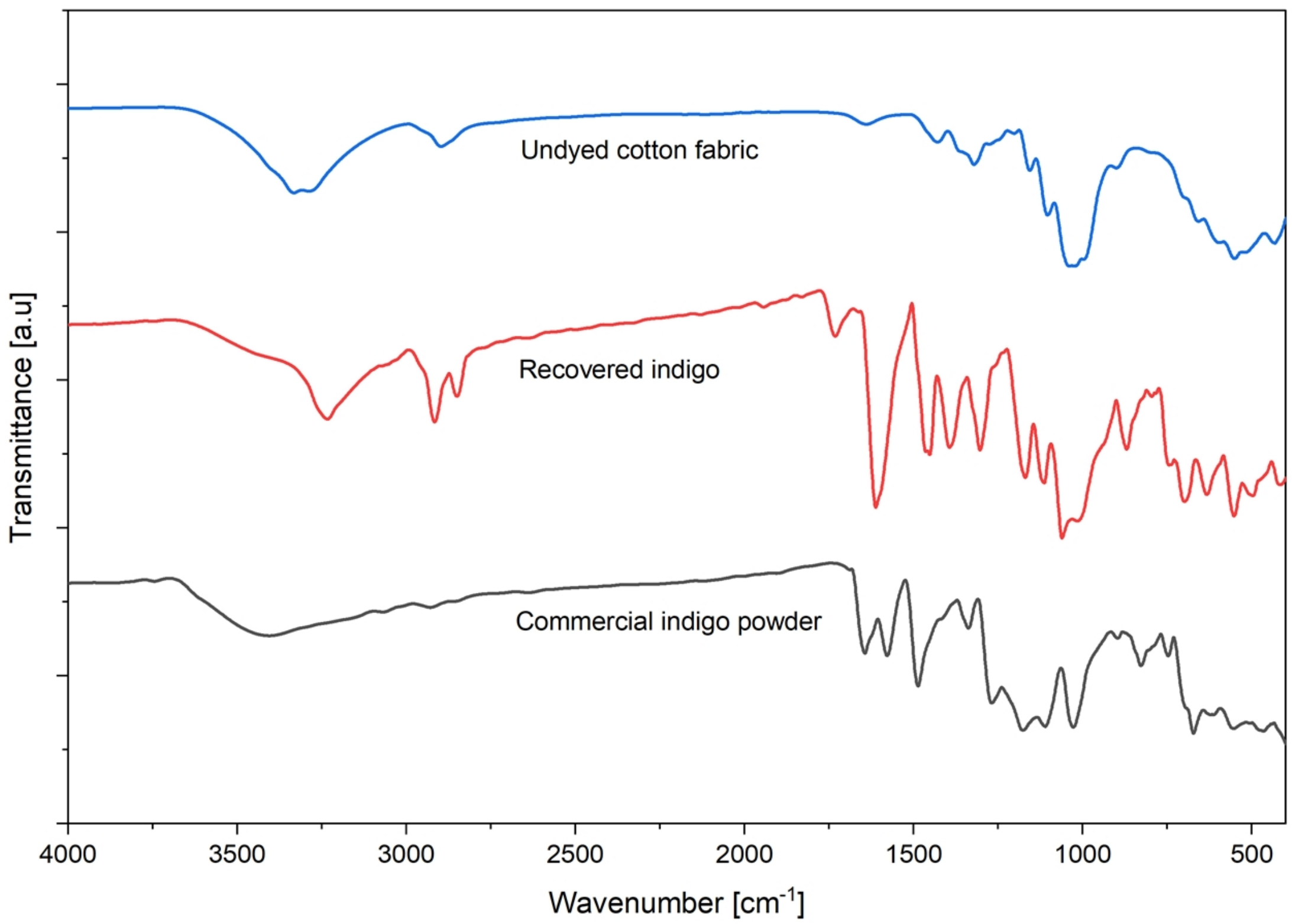

Figure S5. FTIR curve of indigo dye recovered after distillation, commercial indigo powder and undyed cotton fabric.

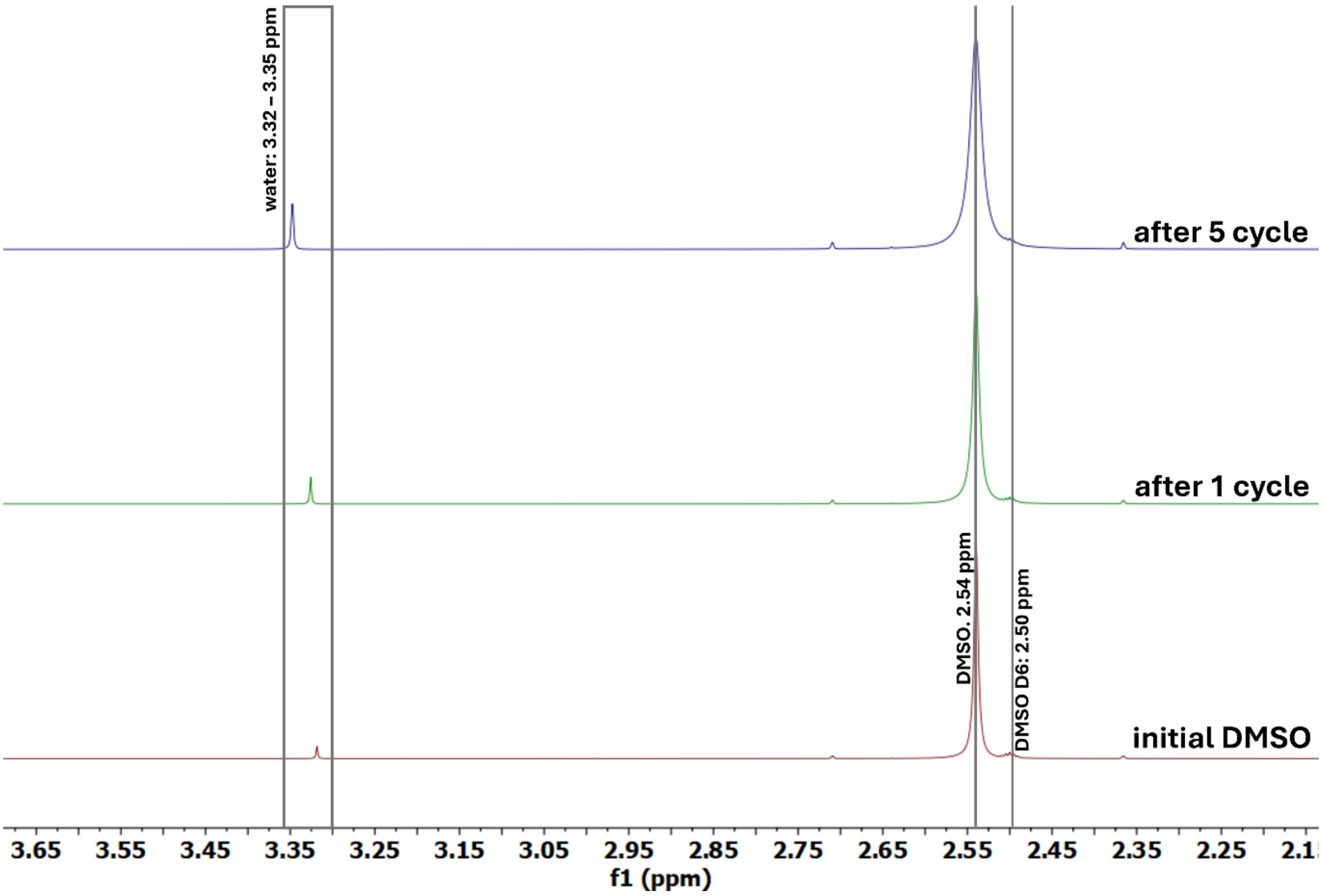

Figure S6. NMR analysis of the three solutions recovered through distillation.

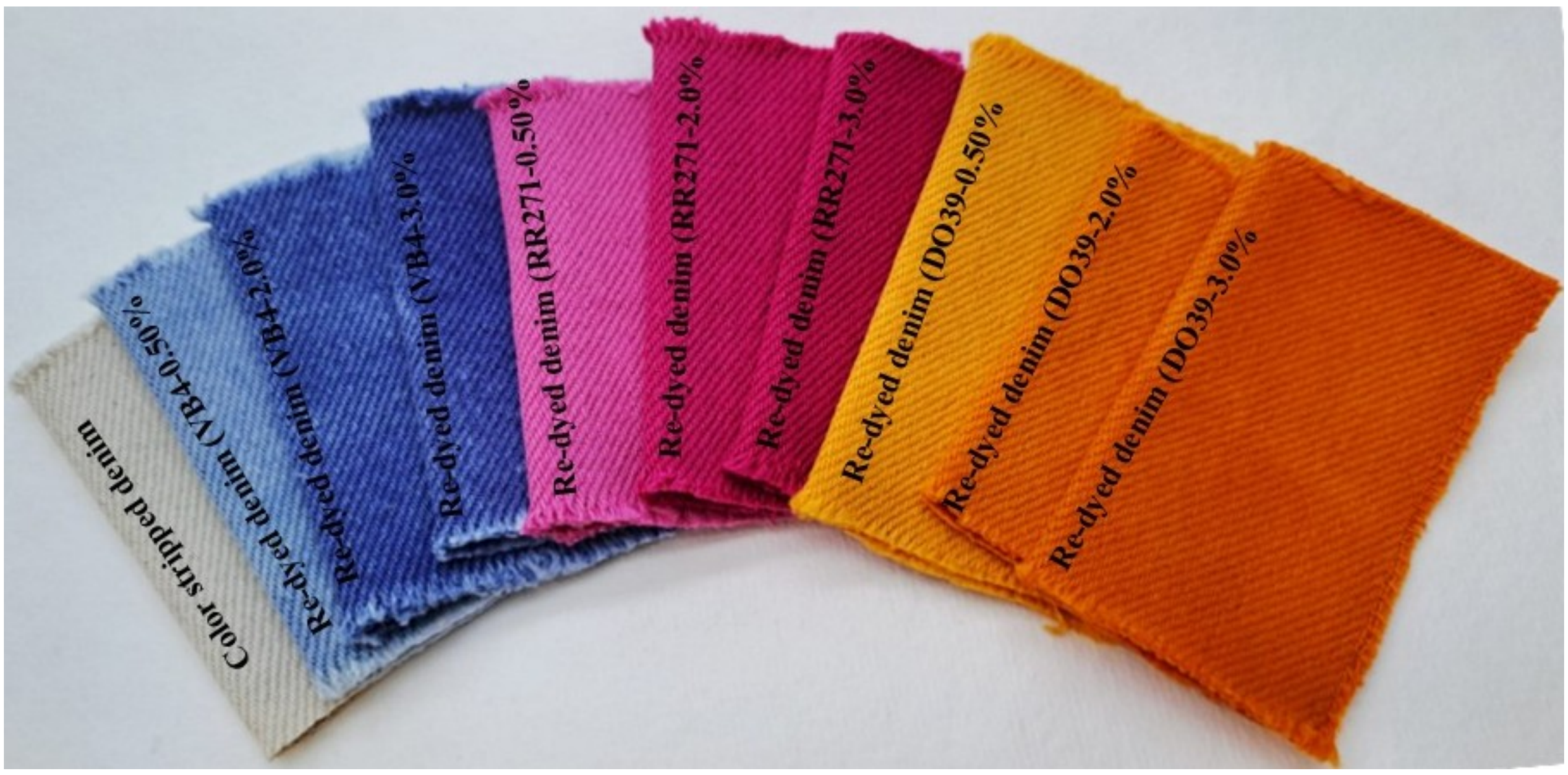

Figure S7. Images of re-dyed denim waste with different commercial dyes (Vat Blue 4, Reactive Red 271 and Direct Orange 39) and shade percentage.

Supplementary tables (S1-S5):

Table S1: NMR Integrals of the DMSO and residual water peaks, along with the calculated water content (%).

	Int. DMSO	Int. water	% of water
initial	1	0.06	6
after 1 cycle	1	0.09	8
after 5 cycle	1	0.13	12

Table S2. Crystallinity Index (%) of dyed denim waste, and color-stripped fabrics.

Sample coding	CI [%]
Color stripped (cycle 3)	47 ± 1
Color stripped (cycle 2)	47 ± 2
Color stripped (cycle 1)	47 ± 2
Dyed denim waste	47 ± 2

Table S3. Onset, peak, endset temperatures, and residual mass values of dyed and color-stripped denim fabrics obtained from TG/DTG analysis

Samples	T_{onset} (°C)	T_{max} (°C)	T_{endset} (°C)	Residual mass (%) at 600°C
Denim_Dyed	343	366	387	7
Denim_Cycle 1	346	367	387	8
Denim_Cycle 2	346	367	383	9
Denim_Cycle 3	345	367	384	9

Table S4. CIE L*a*b* space of redyed color-stripped denim.

Dye type	Shade, %	L*	a*	b*	C*	h°
VB4	0.5	55.45	-4.23	-19.47	19.93	257.74
	2.0	37.45	1.85	-28.66	28.72	273.69
	3.0	32.52	4.32	-28.29	28.62	278.69
RR271	0.5	54.97	35.9	-8.24	36.84	347.07
	2.0	40.29	47.35	-6.08	47.73	352.69
	3.0	38.01	48.39	-4.39	48.59	354.82
DO39	0.5	61.49	28.84	55.65	62.68	62.61
	2.0	50.09	38.76	53.09	65.73	53.87
	3.0	47.82	39.21	51.33	64.59	52.62

Table S5. Suggested interpretation of ΔEcmc values.

ΔEcmc values	Visual appearance of levelness	Extent of unlevelness
≤ 0.20	Excellent levelness	Unlevelness not detectable
0.21-0.50	Good levelness	Unlevelness noticeable under close examination
0.51-1.0	Poor levelness	Apparent unlevelness
> 1.0	Bad levelness	Conspicuous unlevelness
